# Oriented Growth of In‐Oxo Chain Based Metal‐Porphyrin Framework Thin Film for High‐Sensitive Photodetector

**DOI:** 10.1002/advs.202100548

**Published:** 2021-05-17

**Authors:** Yi‐Bo Tian, Nina Vankova, Peter Weidler, Agnieszka Kuc, Thomas Heine, Christof Wöll, Zhi‐Gang Gu, Jian Zhang

**Affiliations:** ^1^ State Key Laboratory of Structural Chemistry Fujian Institute of Research on the Structure of Matter Chinese Academy of Sciences Fuzhou 350002 P. R. China; ^2^ Faculty of Chemistry and Food Chemistry Technical University Dresden Dresden 01069 Germany; ^3^ Institute of Functional Interfaces (IFG) Karlsruhe Institute of Technology (KIT) Eggenstein‐Leopoldshafen 76344 Germany; ^4^ Institute of Resource Ecology Helmholtz‐Center Dresden‐Rossendorf Leipzig Research Branch Leipzig 04318 Germany; ^5^ Fujian Science and Technology Innovation Laboratory for Optoelectronic Information of China Fuzhou Fujian 350108 P. R. China; ^6^ University of Chinese Academy of Sciences Beijing 100049 P. R. China

**Keywords:** In‐oxo chains, metal–organic frameworks, metal‐porphyrin, oriented growth, photodetectors

## Abstract

The potential of metal–organic frameworks (MOFs) for applications in optoelectronics results from a unique combination of interesting photophysical properties and straightforward tunability of organic and inorganic units. Here, it is demonstrated that using MOF approach chromophores can be assembled into well‐ordered 1D arrays using metal‐oxo strands as lead structure, and the resulting porphyrinic rows exhibit unique photophysical properties and allow the realization of highly sensitive photodetectors. A porphyrinic MOF thin film, In‐TCPP surface‐coordinated MOF thin films with [021] orientation is fabricated using a layer‐by‐layer method, from In(NO_3_)_3_ and TCPP (5,10,15,20‐(4‐carboxyphenyl)porphyrin). Detailed experimental and theoretical analysis reveals that the assembly yields a structure where In‐oxo strands running parallel to the substrate fix the chromophoric linkers to yield 1D arrays of porphyrins. The frontier orbitals of this highly anisotropic arrangement are localized in these columnar arrangements of porphyrins and result in high photoactivity, which is exploited to fabricate a photodetector with record (as compared to other organic materials) responsivity in visible regime of 7.28 × 10^14^ Jones and short rise/fall times (0.07/0.04 s). This oriented MOF thin film‐based high‐sensitive photodetector provides a new avenue to use inorganic, stable lead structures to assemble organic semiconductors into regular arrays, thus creating a huge potential for the fabrication of optoelectronic devices.

## Graphical abstract

Highly photoactive, porphyrinic metal–organic framework (MOF) thin films are grown on substrates using layer‐by‐layer process. The unusual architecture of this particular MOF consists of In‐oxo chains acting as lead structure to obtain highly ordered 1D arrays of porphyrins. The MOF thin film is successfully integrated in a visible light photodetector with record sensitivity, 7.28 × 10^14^ Jones.

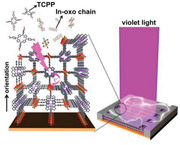

## 1 Introduction

Photodetectors convert photons to electrical signals and are the crucial elements in application fields like biomedical imaging,^[^ ^1^ ^]^ optical communications,^[^ ^2^, ^3^ ^]^ environmental monitoring,^[^ ^4^ ^]^ gas sensing,^[^ ^5^ ^]^ and motion detection.^[^ ^6^ ^]^ Extensive efforts have been devoted to explore next‐generation photodetector materials, including inorganic semiconductors (such as GaN,^[^ ^7^ ^]^ ZnS,^[^ ^8^ ^]^ In_2_Se_3_,^[^ ^9^ ^]^ graphene,^[^ ^10^ ^]^ MoS_2_^[^ ^11^ ^]^) and organic materials.^[^ ^12^, ^13^, ^14^ ^]^ Inorganic semiconductors show excellent performance, but often the fabrication process is complicated and expensive. Organic materials, on the other hand, offer low‐cost production. However, in many cases their responsiveness and stability are inferior in practical photodetection applications. The investigation of hybrid materials, containing inorganic as well as organic constituents, thus appears to be of pronounced interest as synergistic effects may result in superior performance.

Metal–organic frameworks (MOFs) are a prototypic class of such hybrid materials, yet highly ordered compounds. These crystalline networks are fabricated by coordinating organic linkers to metal nodes. While originally MOFs were primarily considered for applications in gas storage/separation,^[^ ^15^, ^16^, ^17^, ^18^, ^19^ ^]^ more recently these reticular materials have found applications in energy storage,^[^ ^20^ ^]^ catalysis,^[^ ^21^, ^22^, ^23^ ^]^ drug delivery,^[^ ^24^, ^25^ ^]^ and water harvesting.^[^ ^26^ ^]^ For many applications requiring electrical contacts and/or exploitation of MOF optical properties,^[^ ^27^ ^]^ MOF thin films are required.^[^ ^28^, ^29^, ^30^, ^31^ ^]^ Indeed, recent work demonstrated that monolithic, uniform, and compact MOF thin films show excellent photoresponse photoconductive and photodetector applications. One example is a porphyrinic MOF, where the donor–acceptor interaction with fullerene guest enhanced charge separation and yielded high photocurrent.^[^ ^32^ ^]^ Erbe and co‐workers reported a photodetector based on a 2D graphene‐like MOF thin film with excellent response over a broad range of wavelengths.^[^ ^33^ ^]^ However, compared to inorganic compounds with detectivities as large as 10^12^–10^15^ Jones^[^ ^34^, ^35^, ^36^ ^]^ and a recently reported molecular crystal with a detectivity for photons in the UV of 10^18^ Jones,^[^ ^37^ ^]^ the sensitivity of MOFs is still substantially smaller, the main reasons being small photocurrents and low photoresponse, with detectivities limited so far to less than 3 × 10^11^ Jones.^[^ ^38^ ^]^

**Scheme 1:**
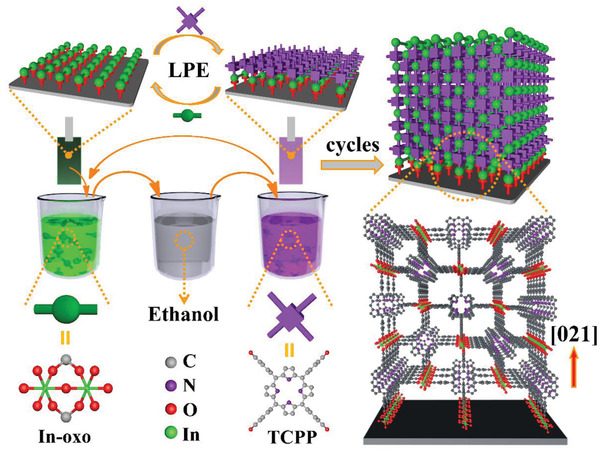
Schematic illustration of In‐TCPP SURMOF grown on functionalized substrate by using layer‐by‐layer dipping method.

In this work, we demonstrate that In‐TCPP, a porphyrinic MOF with a highly anisotropic architecture, is well suited for the construction of photodetectors with detectivities exceeding those reported previously. Whereas most MOFs consist of individual (0D) metal‐oxo nodes linked by organic linkers, in the In‐TCPP 1D MOF metal‐oxo strands provide lead structures to which planar chromophores, TCPP are fixed via coupling units so as to well‐ordered rows running along the [100] direction (**Scheme** **1**). The choice of In‐oxo strands was motivated by the attractive properties of In_2_O_3_ with regard to the fabrication of optical sensors and devices.^[^ ^39^, ^40^ ^]^ The chromophores used here, porphyrins, exhibit excellent electron‐transfer^[^ ^41^ ^]^ and photoresponse properties and are often used in organic photovoltaic devices.^[^ ^42^ ^]^

With regard to integrating photoactive materials into devices, the fabrication of thin films is required, and in addition achieving oriented growth is either required or beneficial.^[^ ^43^ ^]^ As thin films made from the conventional, powdery form of MOFs obtained from solvothermal synthesis schemes are rather inhomogeneous, we have used a layer‐by‐layer (lbl) method for film deposition, which allows growing monolithic MOF thin films with well‐defined thickness. The surface‐coordinated MOF thin films (SURMOFs) exhibit a high degree of orientation, with the 1D porphyrinic columns aligned parallel to the substrate. The interlamellar distance of porphyrinic ligands with face‐to‐face stacking is 7.1 Å, which is close to the interplanar distance in 2D porphyrinic MOFs (6.7 Å) as listed in Table S1 (Supporting Information).

**Figure 1:**
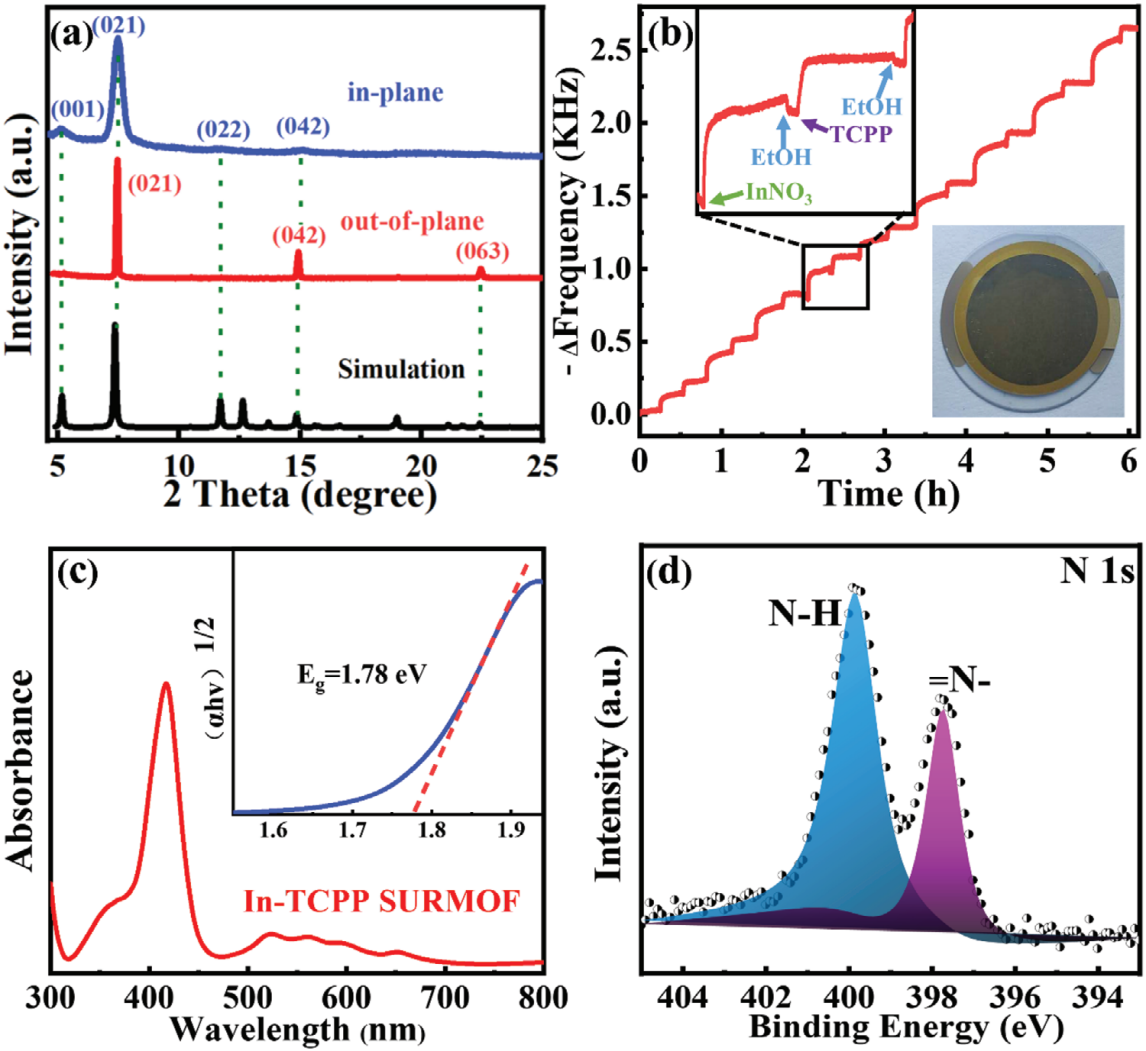
Characterization of In‐TCPP SURMOF: a) out‐of‐plane and in‐plane XRD pattern of In‐TCPP SURMOF and simulated XRD pattern of bulk In‐TCPP; b) the frequency decrease (−Δ*f*) in the lbl growth process monitored by a QCM‐D (inset: sense chip assembled with In‐TCPP SURMOF); c) UV–vis spectra (inset: Tauc plot); d) N 1s region of XPS data.

The liquid phase epitaxial (LPE) lbl dipping method^[^ ^44^, ^45^ ^]^ is illustrated in Scheme 1. Briefly, a functionalized substrate (hydroxyl‐functionalized SiO_2_/Si, for details see the Supporting Information) was immersed subsequently into In(NO_3_)_3_ and TCPP(H_2_) solutions. Between each of these steps the substrate was rinsed with ethanol. For the samples used here, typically 15 preparation cycles were used, yielding uniform In‐TCPP SURMOFs, with a high degree of crystallite orientation and a thickness of about 170 nm. The X‐ray diffraction (XRD) data displayed in **Figure** **1** demonstrate that the In‐oxo chains and the rows of porphyrins running along the [021] crystallographic direction are almost perfectly aligned parallel to the substrate. The stoichiometry of the In‐oxo chain in In‐TCPP is calculated as In(COO)_2_(OH).

The obtained In‐TCPP SURMOF was used to fabricate a two‐terminal photodetector, which showed a very large detectivity (D*) of 7.28 × 10^14^ Jones for a bias of 10 V at 420 nm light irradiation. The performance is much higher than that of reference devices fabricated from powder‐based, polycrystalline In‐TCPP thin films and also higher than other photodetectors based on porphyrinic complexes, organic compounds, perovskites, and reported MOFs (Table S2, Supporting Information). This work demonstrates that the LPE lbl approach offers an enormous potential to fabricate high‐quality SURMOFs containing inorganic In‐oxo chains and organic porphyrinic components for high‐sensitive photodetection.

The out‐of‐plane XRD pattern of the obtained In‐TCPP SURMOF (Figure 1a) shows three sharp diffraction peaks at 7.5°, 15.0°, and 22.5°. By comparing with a simulation using the previously reported structure of the In‐TCPP MOF,^[^ ^46^ ^]^ these signals can be assigned to the (021), (042), and (063) reflections. The in‐plane XRD data show distinct peaks at 5.2°, 7.5°, 11.7°, and 15.0°, which are related to the (001), (021), (022), and (042) peaks. Some weak diffraction peaks are submerged in the noise baseline owing to the very thin of SURMOF. The intense peaks in the out‐of‐plane and in‐plane XRD patterns as well as the small width of the individual peaks clearly demonstrate the presence of highly crystalline In‐TCPP SURMOF with the [021]‐direction oriented perpendicular to the substrate. For comparison, polycrystalline In‐TCPP thin films were prepared from solvothermally synthesized powders and also characterized by XRD and scanning electron microscopy (SEM) (Figures S1 and S9, Supporting Information). A quartz crystal microbalance (QCM) was used to in situ monitor the lbl growth process of In‐TCPP SURMOF by recording the frequency and dissipation (QCM‐D) changes of a modified Au chip sensor surface successively exposed to In(NO_3_)_3_ and TCPP solutions (Figure 1b and Figure S2, Supporting Information). During the growth‐monitoring experiments, In(NO_3_)_3_ and TCPP solutions were alternately pumped through the QCM‐D cell with ethanol washing between each step. The frequency change was plotted against the number of pumping lbl cycles in Figure 1b. The flowing rate of pump was constant during the experiment. Growth was initiated by introduction of ethanolic In(NO_3_)_3_, which caused a sharp rise in minus frequency (−Δ*f*) that reached a plateau after ≈15 min. Then ethanol was passed over the QCM‐D cell, leading a slight drop in minus frequency, presumably owing to the dissociation of unreacted species. Afterward, TCPP solution was flowed over the QCM‐D cell, which was again accompanied by a significant increase of minus frequency. The species deposited can be indirectly determined from the mass gain/cycle and the density of reactive sites per unit area.^[^ ^47^ ^]^ UV–vis spectra (Figure 1c) recorded for In‐TCPP SURMOF show a strong absorption band at around 420 nm, demonstrating an excellent photoresponse to visible light. In the range of 500–700 nm four Q‐band characteristic peaks are observed, demonstrating that the porphyrinic groups are not protonated.^[^ ^48^ ^]^ In contrast, the UV–vis spectra of the In‐TCPP thin film made from solvothermally synthesized powder material (Figure S3, Supporting Information) show a broad absorption band from UV to visible light, indicating that a certain percentage of the porphyrins were metalated during the high‐temperature (120 °C) synthesis. The N 1s X‐ray photoelectron spectroscopy (XPS) peaks (Figure 1d) of In‐TCPP SURMOF at 399.8 and 397.7 eV are ascribed to the N—H and =N— of free‐based porphyrinic units, which are similar to the N 1s XPS data recorded for the TCPP ligand (Figure S4, Supporting Information). Figure S5 (Supporting Information) shows In 3d XPS peaks at 452.8 and 445.3 eV, demonstrating the valence of In cation in In‐TCPP is +3. The analysis of the infrared reflection absorption spectroscopy (IRRAS) data for the In‐TCPP SURMOF (Figure S6, Supporting Information) is fully consistent with a coordination of four carboxylates to the In‐oxo chains.

**Figure 2:**
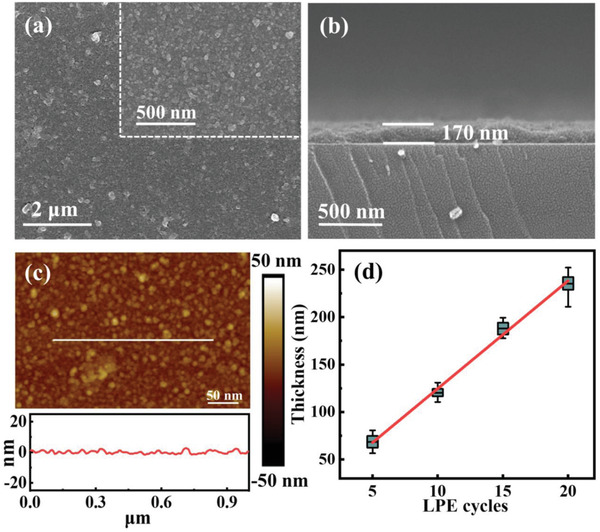
a) Surface SEM image, b) cross‐sectional SEM image, and c) AFM image with roughness statistics of In‐TCPP SURMOF with 15 cycles; d) the thickness of In‐TCPP SURMOF with 5, 10, 15, and 20 LPE cycles.

The surface (**Figure** **2**a) and cross‐sectional scanning electron microscopy (SEM) images (Figure 2b) demonstrate that the In‐TCPP SURMOFs prepared by LPE lbl process are homogeneous, compact, and continuous. By varying the number of deposition cycles, In‐TCPP SURMOFs with thicknesses (as determined from cross‐sectional SEM images, Figure 2b and Figure S7, Supporting Information) of ≈60, ≈130, ≈170, and ≈240 nm were fabricated, using 5, 10, 15, and 20 cycles, respectively. Atomic force microscopy (AFM) images and the corresponding roughness analysis (Figure 2c) reveal rather flat surfaces of the MOF thin films with a mean roughness of ≈5 nm. The contact angle measurements (Figure S8, Supporting Information) reveal a pronounced hydrophobic character of the MOF thin film, a beneficial property with regard to device fabrication. In contrast, the SEM images of powdery In‐TCPP thin films (Figure S9, Supporting Information) show a rough and noncompact surface.

**Figure 3:**
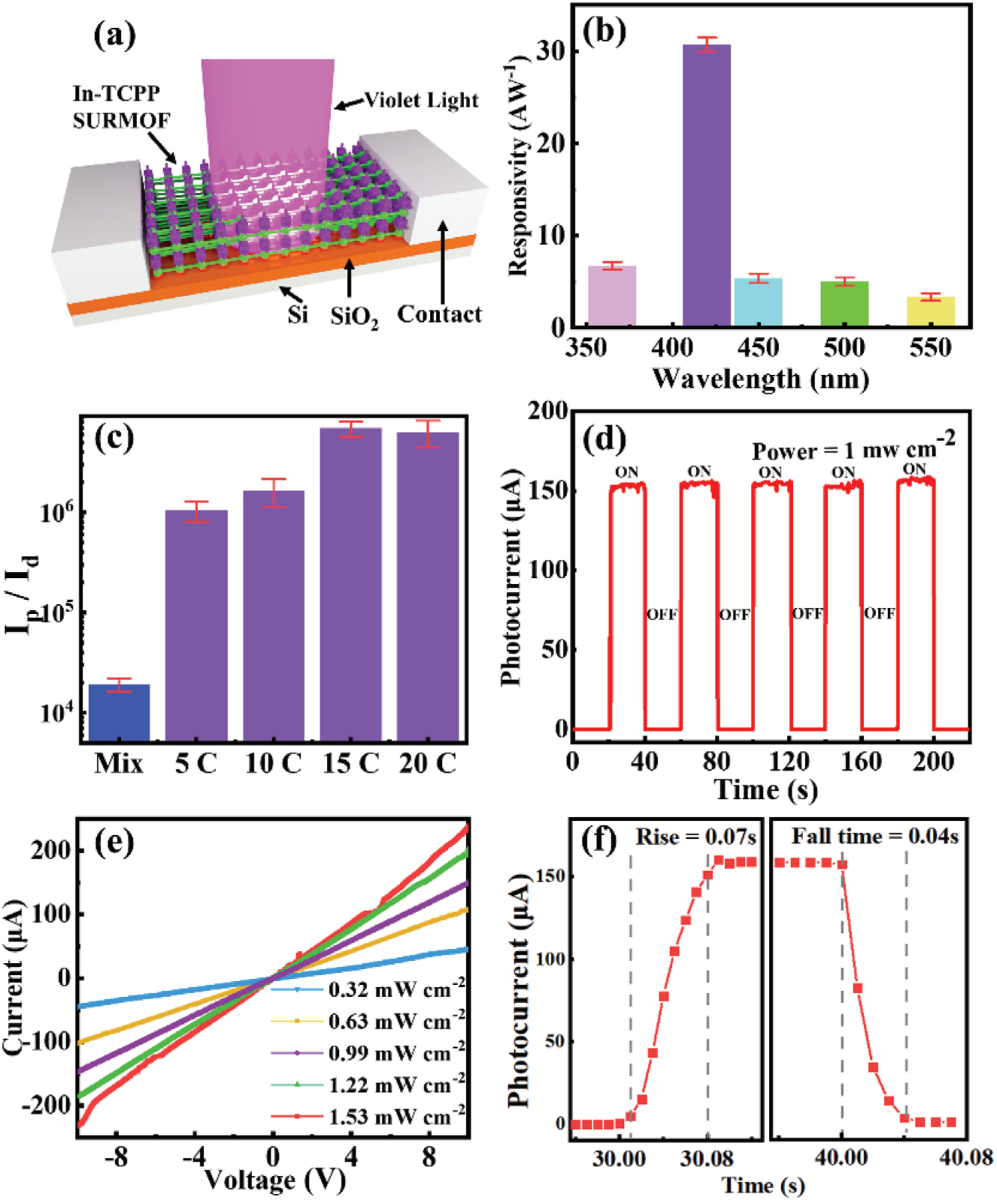
a) Schematic diagram of In‐TCPP SURMOF‐based photodetector; b) responsivities versus light absorption of In‐TCPP SURMOF at different wavelengths pulses irradiation; c) the on/off ratio of mix‐oriented In‐TCPP thin film and In‐TCPP SURMOF with different thickness; d) time‐resolved photocurrent as a function of illumination wavelength at 10 V bias voltage; e) light intensity dependent *I*–*V* curves of In‐TCPP SURMOF; and f) the rise/fall times curve for In‐TCPP SURMOF illuminated with 420 nm light at 10 V bias voltage.

The performances of In‐TCPP SURMOF‐based photodetectors were studied by using a commercial semiconductor analysis system using the two‐probe method as illustrated in **Figure** **3**a. The effective area of the photodetector amounts to 0.01 cm^2^. Silver with low work function is used as electrode contact. To evaluate the device performance, the responsivity (*R*_*λ*_) is determined in the wavelength regime of 365–600 nm (Figure 3b). These data reveal that the optimum responsivity of In‐TCPP SURMOF is at 420 nm, which is consistent with the UV–vis absorption band and the wavelength‐dependent photocurrent (Figure S10, Supporting Information). Figure 3c reveals that In‐TCPP SURMOF‐based photodetector had much higher on/off ratio (6.8 × 10^6^) than the powdery thin film under violet light. The on/off ratio showed a strong dependence on the thickness of In‐TCPP thin film. The photoresponsivity increased with increasing the thickness due to its decrease of electric resistance. For semiconductors, the resistance is inversely proportional to thickness of thin film,^[^ ^49^ ^]^ which is consistent with our test (Table S3, Supporting Information). To a certain extent, In‐TCPP SURMOF with higher thickness displays lower dark current (Figure S11, Supporting Information), which suggests that photogenerated electrons from porphyrinic groups are easier to be conducted and monitored. Furthermore, the on/off ratio of In‐TCPP SURMOF with 20 cycles slightly lower than that of In‐TCPP SURMOF with 15 cycles (Table S4 and Figure S12, Supporting Information). In our experiments, the highest sensitivity for violet light (420 nm) was observed for an In‐TCPP SURMOF with a thickness of ≈170 nm (15 cycles), and accordingly films with this thickness were used for the realization of a photodetector. As shown in Figure 3d and Figure S13 (Supporting Information), this In‐TCPP SURMOF device exhibited a stable and reversible on/off photocurrent when irradiated with periodic light pulses. Under 1 mW cm^−2^ of 420 nm irradiation, the responsivity (*R*_*λ*_) and detectivity (D*) of this photodetector are calculated to be 30.8 A W^–1^ and 7.28 × 10^14^ Jones at a bias of 10 V, respectively. The *I*–*V* test result (Figure 3e) displays the response current of the detector increases with the rising irradiation intensity. The resulting In‐TCPP SURMOF‐based photodetector is operated in photoconductive mode. Figure 3f shows In‐TCPP SURMOF‐based photodetector has a short rise/fall time with the value of 0.07/0.04 s, which is shorter than that of most reported organic and MOFs‐based photodetectors.^[^ ^33^ ^]^ This can be attributed that the In‐oxo chains provide a fast transport path for the carrier transport and reduce the recombination possibility of the photoinduced charge carries, resulting in a fast photoresponse in the In‐TCPP SURMOF.

**Figure 4:**
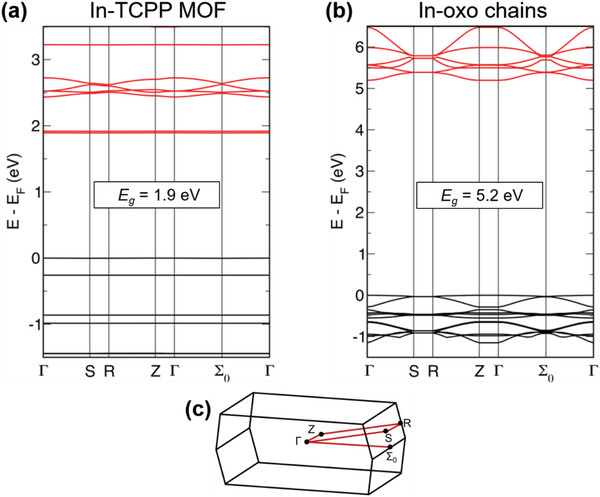
Band structure of a) In‐TCPP MOF and b) In‐oxo‐based chain model, and c) representation of the primitive Brillouin zone with the calculated path Γ→S→R→Z→Γ→Σ_0_→Γ. Calculations with Crystal17 at the PBE‐D3(BJ)/POB‐TZVP level of theory. Structural models used in the calculations are represented in Figures S15–S16 (Supporting Information).

To understand the mechanism for the efficacy of photoexcited charge carrier conduction in In‐TCPP SURMOF system, density functional theory (DFT) calculations were carried out (details see the Supporting Information) using the structural unit cell as the calculation model. The electronic bands of In‐TCPP are displayed in **Figure** **4**a and reveal the presence of a direct bandgap with 1.89 eV. While this value agrees well with that determined from the Tauc plot of the experimental data, it must be noted that (i) the very flat nature of both conduction and valence bands do not allow a clear assignment of the bandgap character, and (ii) the used gradient‐corrected (PBE) density functional generally underestimates optical gaps, as reflected in the hybrid functional (HSE06) calculations shown in Figure S14 (Supporting Information). However, it does not account for exciton binding energies which may be appreciable for MOF materials owing to their low dielectric constant.^[^ ^50^ ^]^

As demonstrated by the integrated density of states (IDOS) (Figure S17, Supporting Information), the frontier bands are both dominated by the porphyrinic units. Given the fact that both bands are extremely flat, there is negligible conjugation between neighboring molecules. Therefore, band transport is not possible, and the charge transport is deduced to be due to hopping transport along the porphyrin rows. Note, that charge transport should be rather efficient along the porphyrin chains because of the high degree of order compared with other forms of condensed porphyrins. Of course, in a photodetector material, in addition to high cross sections for photon absorption, charge separation is required. Obviously, the band structure for the perfect In‐TCPP as shown in Figure 4 does not provide a mechanism for exciton dissociation. However, the In‐oxo chains are expected to exhibit defects (e.g., O‐vacancies), resulting in donor and acceptor states. As the defect density is too low, it is impossible to see them directly in the UV–vis experiments. However, the rather large exciton diffusion length along the highly ordered porphyrin chains is expected to result in a large probability that photogenerated excitons reach a defect for charge separation, thus explaining the large detectivities observed in our experiments.

## 2 Conclusions

In summary, this work first reports a proof‐of‐concept photodetector assembled from an In‐oxo chain‐based metal‐porphyrin framework thin film. By using a layer‐by‐layer strategy, monolithic, highly homogeneous In‐TCPP SURMOFs with their [021]‐orientation perpendicular to the substrate were grown on functionalized substrates, with In‐oxo chains running parallel to the substrate. These oxidic, rigid chains assemble the chromophoric linkers into highly ordered 1D arrays of porphyrins. The parameters used to fabricate photodetectors from this material were optimized, yielding a porphyrin‐based In‐TCPP SURMOF with record detectivity (D*) of 7.28 × 10^14^ Jones and short rise/fall times (0.07/0.04 s), thus outperforming other porphyrinic and chromophoric systems in the visible regime.

DFT calculations demonstrate that the frontier orbitals of this highly anisotropic arrangement are localized in these columnar arrangements of porphyrins and suggest that the hopping transport along these highly ordered rows provided the basis for the high photodetection performance. Our findings not only provide a new type of oriented MOF thin film material well‐suited for the fabrication of highly sensitive photodetectors in the visible regime but also open up an attractive route to use inorganic metal‐oxo chains as lead structure to yield highly ordered 1D arrays of organic ligands for advanced optoelectronic applications.

## Conflict of Interest

The authors declare no conflict of interest.

## Supporting information

Supporting Information

## Data Availability

Research data are not shared.
