# Supplementary material for: Oriented Growth of In‐Oxo Chain Based Metal‐Porphyrin Framework Thin Film for High‐Sensitive Photodetector
Source: Adv Sci (Weinh). 2021 May 17;8(14):2100548. doi: 10.1002/advs.202100548 (PMC8292912; doi:10.1002/advs.202100548)
Supplement: Supplementary file 1 — Supporting Information [file ADVS-8-2100548-s001.pdf]

© 2021 Wiley-VCH GmbH

ADVANCED SCIENCE Open Access

Supporting Information

for *Adv. Sci.*, DOI: 10.1002/advs.202100548

Oriented Growth of In-Oxo Chain based Metal-Porphyrin Framework Thin Film for High-Sensitive Photodetector

Yi-Bo Tian, Nina Vankova, Peter Weidler, Agnieszka Kuc, Thomas Heine, Christof Wöll, Zhi-Gang Gu and Jian Zhang*

Supporting Information

Oriented Growth of In-Oxo Chain based Metal-Porphyrin Framework Thin Film for High-Sensitive Photodetector

Yi-Bo Tian, Nina Vankova, Peter Weidler, Agnieszka Kuc, Thomas Heine, Christof Wöll, Zhi-Gang Gu and Jian Zhang*

Keywords: metal-organic framework, oriented growth, In-oxo chain, metal-porphyrin, photodetector

Table of Contents

**Experimental Procedures**
**Materials and Instruments**
**Fabrication of In-TCPP SURMOF and mix-oriented thin film**
***in-situ* growth process of In-TCPP SURMOF**
**Fabrication of In-TCPP based photodetector**
**Computational details**
**Figure S1.** XRD pattern of mix-oriented In-TCPP thin film (red) and simulated XRD pattern of bulk In-TCPP (black).
**Figure S2.** QCM-D results showing dissipation changes after $In(NO_3)_3$ and TCPP deposition cycles with EtOH rinsing on Au-coated electrodes at 50 °C. The flow rate was maintained constant at 100 µL/min during the experiment.
**Figure S3.** UV-vis spectra of mix-oriented In-TCPP thin film.
**Figure S4.** The N 1s XPS spectra of TCPP ligand.
**Figure S5.** The In 3d XPS spectra of In-TCPP SURMOF.
**Figure S6.** The IRRAS spectra of In-TCPP SURMOF and TCPP ligand.
**Figure S7.** SEM images for different cycles of In-TCPP SURMOF on the SiO_2/Si substrate (a) 5 cycles, (b) 10 cycles and (c) 20 cycles.
**Figure S8.** The contact angles of bare SiO_2/Si substrate (a) and In-TCPP SURMOF (b).
**Figure S9.** SEM images of mix-oriented In-TCPP thin film.
**Figure S10.** Wavelength-dependent photoresponse of In-TCPP SURMOF based photodetector.
**Figure S11.** I-t curve of In-TCPP SURMOF with different thickness and mix-oriented thin film: (a) mix-oriented thin film, (b) 5 cycles, (c) 10 cycles and (d) 20 cycles
**Figure S12.** AFM image with roughness for In-TCPP SURMOF with 20 cycles.
**Figure S13.** Time-resolved photocurrent stability test of light and dark current under 420 nm, each round of illumination time is 60 s.
**Figure S14.** Band structure of In-TCPP MOF (a) and of In-oxo based chain model (b), and representation of the primitive Brillouin zone with the calculated path

$\Gamma \rightarrow S \rightarrow R \rightarrow Z \rightarrow \Gamma \rightarrow \Sigma_0 \rightarrow \Gamma$ (c). Calculations with Crystal17 at the HSE06-D3(BJ)/POB-TZVP//PBE-D3(BJ)/POB-TZVP level of theory.
**Figure S15.** Atomistic representation of the In-TCPP MOF along the different crystallographic directions. The quadrilaterals indicate the unit cell used in the calculations. Color code: In orange, O red, N blue, C grey, H white.
**Figure S16.** Atomistic representation of the In-oxo based chain model along the different crystallographic directions. Color code: In orange, O red, C grey, H white.
**Figure S17.** Integrated Density-of-States per projection (IDOS), as calculated for the In-TCPP MOF (a) and for the In-oxo based chain model (b). VB denotes valence band maximum, CB denotes conduction band minimum. Calculations with Crystal17 at the PBE-D3(BJ)/POB-TZVP//PBE-D3(BJ)/POB-TZVP level of theory.
**Table S1.** Comparison of the interlamellar distance of porphyrinic MOFs with face-to-face stacking.
**Table S2.** Comparison of characteristic parameters for state-of-the-art photodetectors
**Table S3.** Resistance of In-TCPP SURMOFs with different LPE cycles.
**Table S4.** The roughness of In-TCPP SURMOF with 15 and 20 cycles recording by AFM.
**References**

Experimental Procedures

Materials and Instruments

In this work, all of the reagents and solvents used were commercially available without any further purification. X-ray diffraction (XRD) analysis was recorded by the MiniFlex 600 with Cu-Kα radiation (λ = 0.1542 nm) in the 2θ range of 5-30 °. The oriented In-TCPP thin film grown on functionalized Au substrate was characterized for the infrared reflection absorption spectroscopy (IRRAS). The IRRAS data was performed on a Bruker Vertex 70 FTIR spectrometer. The UV-vis spectra for samples from 300 to 800 nm were performed on Lambda 365. Scanning electron microscope (SEM) images for the samples were measured by ZEISS Gemini 300 SEM. Transmission electron microscope (TEM) images recorded for the samples were used by JEM-2010F. Atomic force microscopy (AFM) images of the samples were recorded with the Bruker Dimension ICON. The X-ray photoelectron spectroscopy (XPS) spectra for the samples were measured by ESCALAB 250Xi. The *in-situ* growth process of MOF thin film was measured by a quartz crystal microbalance (QCM) Q-Sense Explorer (QSX301, Q-Sense, Sweden). Electrical measurements were performed using Keithely 4200 semiconductor analysis system.

Fabrication of In-TCPP SURMOF and mix-oriented thin film

The In-TCPP SURMOF was established by liquid-phase epitaxial (LPE) layer-by-layer dipping method.[1] The OH-functionalized SiO_2/Si substrates were prepared by using a mixture of 2 mM NaOH solution and 30 % hydrogen peroxide with a ratio 3:1 at 80 °C for 30 min, and then rinsed with the deionized water and dried under nitrogen flux for the next step. The In-TCPP SURMOF was fabricated using the following diluted ethanolic solutions: 1 mM indium nitrate and 0.1 mM TCPP. The immersion times were 20 min for $In(NO_3)_3$ solution and 30 min for TCPP solution, respectively. Each step was followed by a rinsing step with pure ethanol to remove residual reactants. For comparison, 5, 10, 15 and 20 cycles of SURMOFs were prepared in this work. In addition, the mix-oriented thin film was synthesized by mixing Indium nitrate (150 mg, 0.5 mmol), 5,10,15,20-tetra(4-carboxyphenyl) porphyrin

(TCPP) (158 mg, 0.2 mmol) and 10 ml DMF in a sealed glass bottle, and then put the functional substrate into the solution and keep at 120 °C for 1 day. The obtained sample was washed with DMF and ethanol in turn, and dried in vacuum at 60 °C. Finally, the In-TCPP film with mixed orientation was assembled on SiO_2/Si substrate.

***in-situ* monitoring the growth process of In-TCPP SURMOF**

A commercial Q-Sense Explorer QCM-D (Q-Sense, Sweden) with a flow module for measurements in liquid phase was used to monitor the in-situ growth of In-TCPP SURMOF on 11-mercapto-1-undecanol (MUD) SAMs functionalized chip, which were prepared as-received QCM working electrodes. The sensor is AT cut and gold coated with a total diameter of 14 mm, a thickness of 0.3 mm and a basis frequency of 4.95 MHz ± 50 kHz. The working electrode has a diameter of 10 mm. The thickness of the gold layer amounts to 100 nm. The temperature of the QCM-D cell was set to 50 °C and the flow rate was kept at 100 µl/min for $In(NO_3)_3$ (1 mM) solutions and TCPP (0.2 mM, equimolar). Pure ethanol was used for the baseline and rinsing step during the experiments. A decrease of the resonance frequency is due to the increase of the mass on QCM sensor according to the equation $\Delta f = -C\Delta M$, where f is the resonance frequency, M is the mass of crystal and C the mass sensitivity constant is referred to the equation[2, 3]

Fabrication of In-TCPP based photodetector

Silver was adopted as the electrode material to form an ohmic contact with MOF thin film. Copper interdigital electrode grids as a shadow mask was covered on the surface of MOF thin film. Then the samples were patterned with Ag electrodes by using a shadow mask technique under Ag vapors in a thermal evaporation chamber. The effective illuminated area was 0.01 cm^2.

Computational details

First-principle calculations based on density functional theory (DFT) were performed by using the Crystal17 code.[4] POB-TZVP (triple-zeta valence with polarization quality) basis sets,[5] and D3(BJ) dispersion corrections[6] to account for the London dispersion interactions were used throughout the calculations. Relaxation of the atomic positions in both the In-TCPP SURMOF and the In-oxo based chain model was carried out using the Perdew–Burke–Ernzerhof (PBE)[7] GGA functional while keeping the lattice parameters the same as the previously reported crystallographic cell (a = 7.17 Å, b = 32.67 Å and c = 17.06 Å ($\alpha = \beta = \gamma = 90°$).[8] Band structures, projected and integrated density of states (pDOS and IDOS, resp.) were then calculated at the same GGA PBE-D3(BJ) level, but also using the Heyd-Scuseria-Ernzerhof (HSE06) screened hybrid functional[9]. In all calculations, we used a k-point mesh of 52 k-points in the irreducible Brillouin zone according to the Monkhorst-Pack sampling scheme.[10]

Results and Discussion

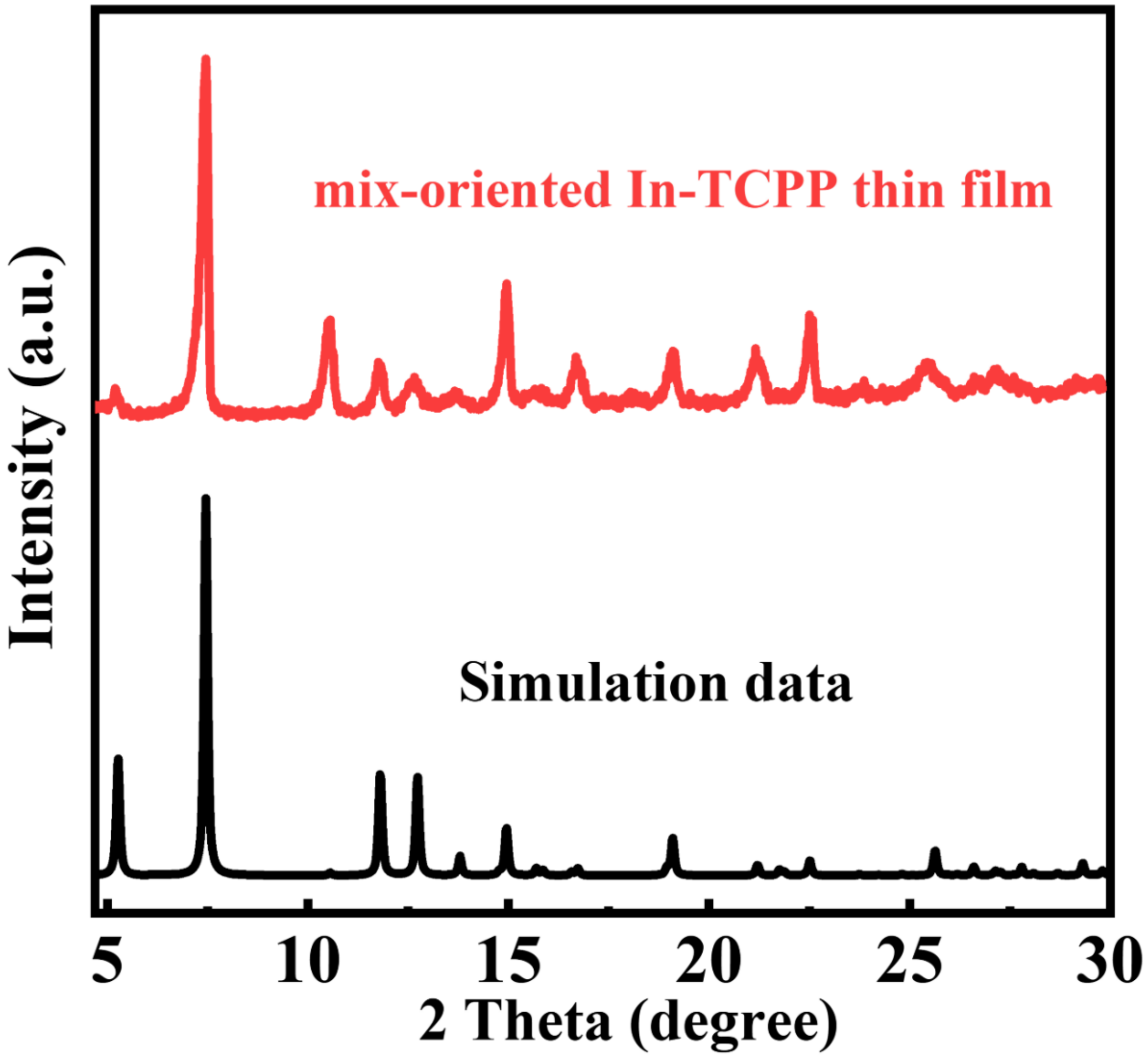

Figure S1. XRD pattern of mix-oriented In-TCPP thin film (red) and simulated XRD pattern of bulk In-TCPP (black).

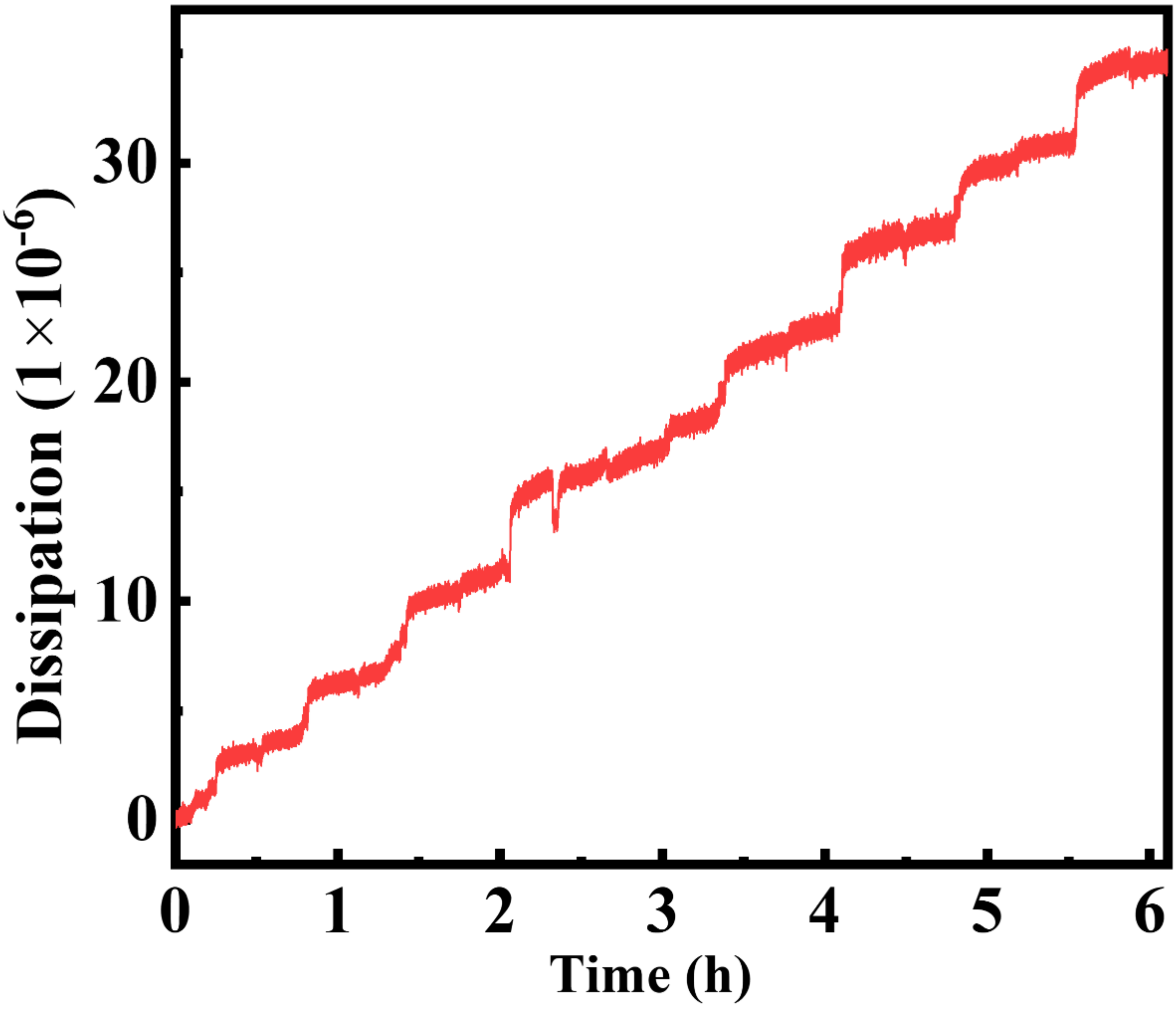

Figure S2. QCM-D results showing dissipation changes after $In(NO_3)_3$ and TCPP deposition cycles with EtOH rinsing on Au-coated electrodes at 50 °C. The flow rate was maintained constant at 100 μL/min during the experiment.

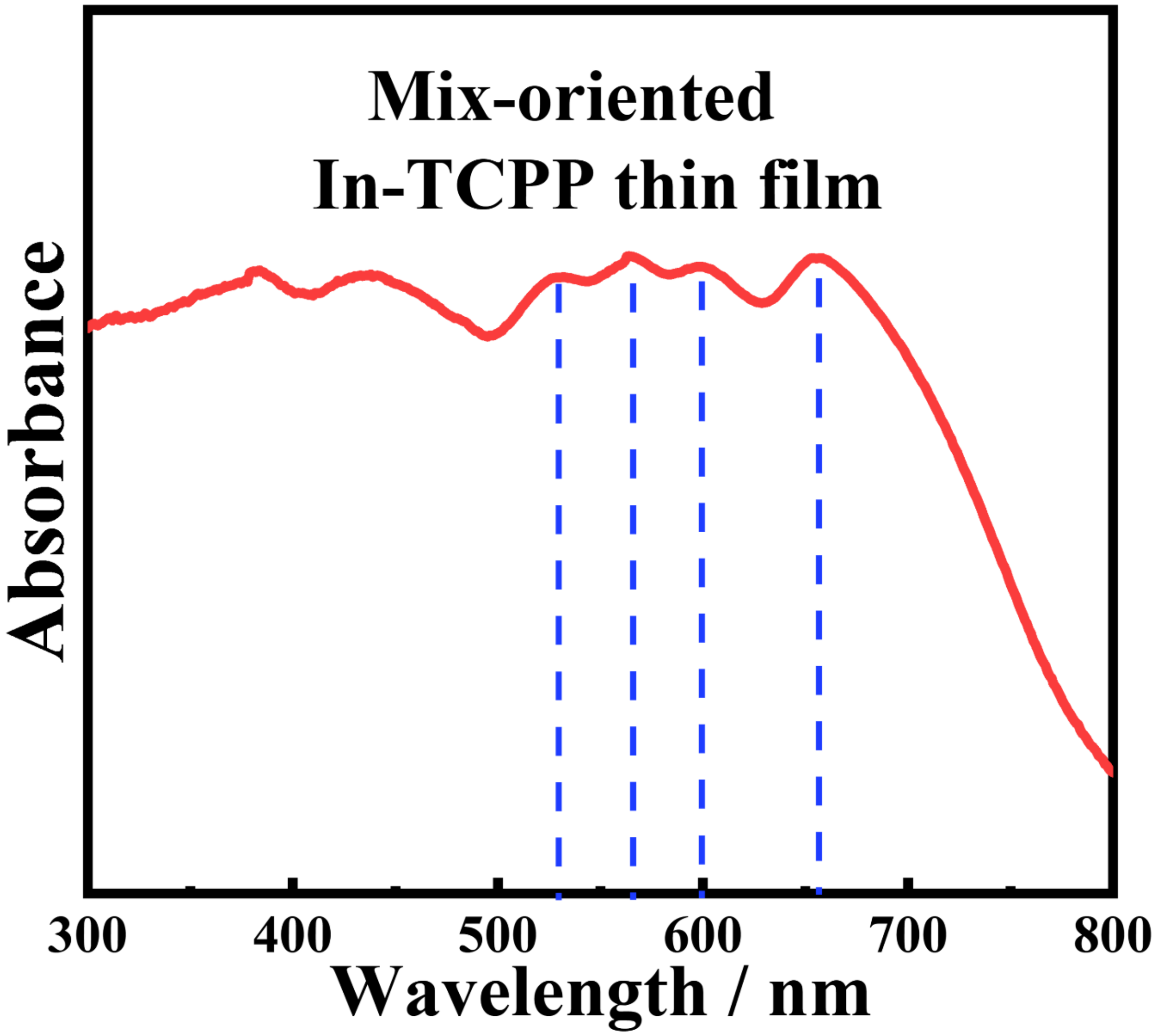

Figure S3. UV-vis spectra of mix-oriented In-TCPP thin film.

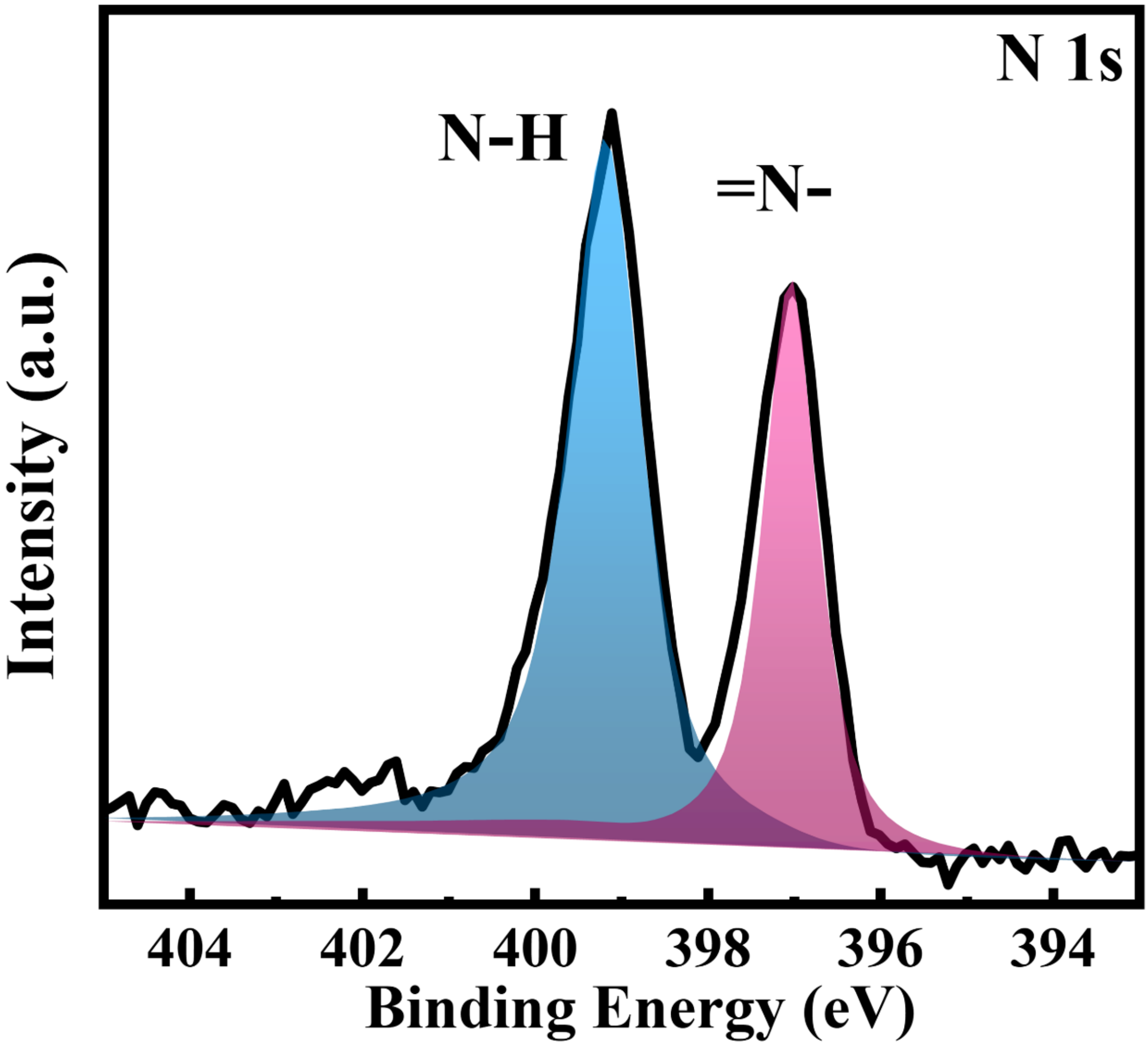

Figure S4. The N 1s XPS spectra of TCPP ligand.

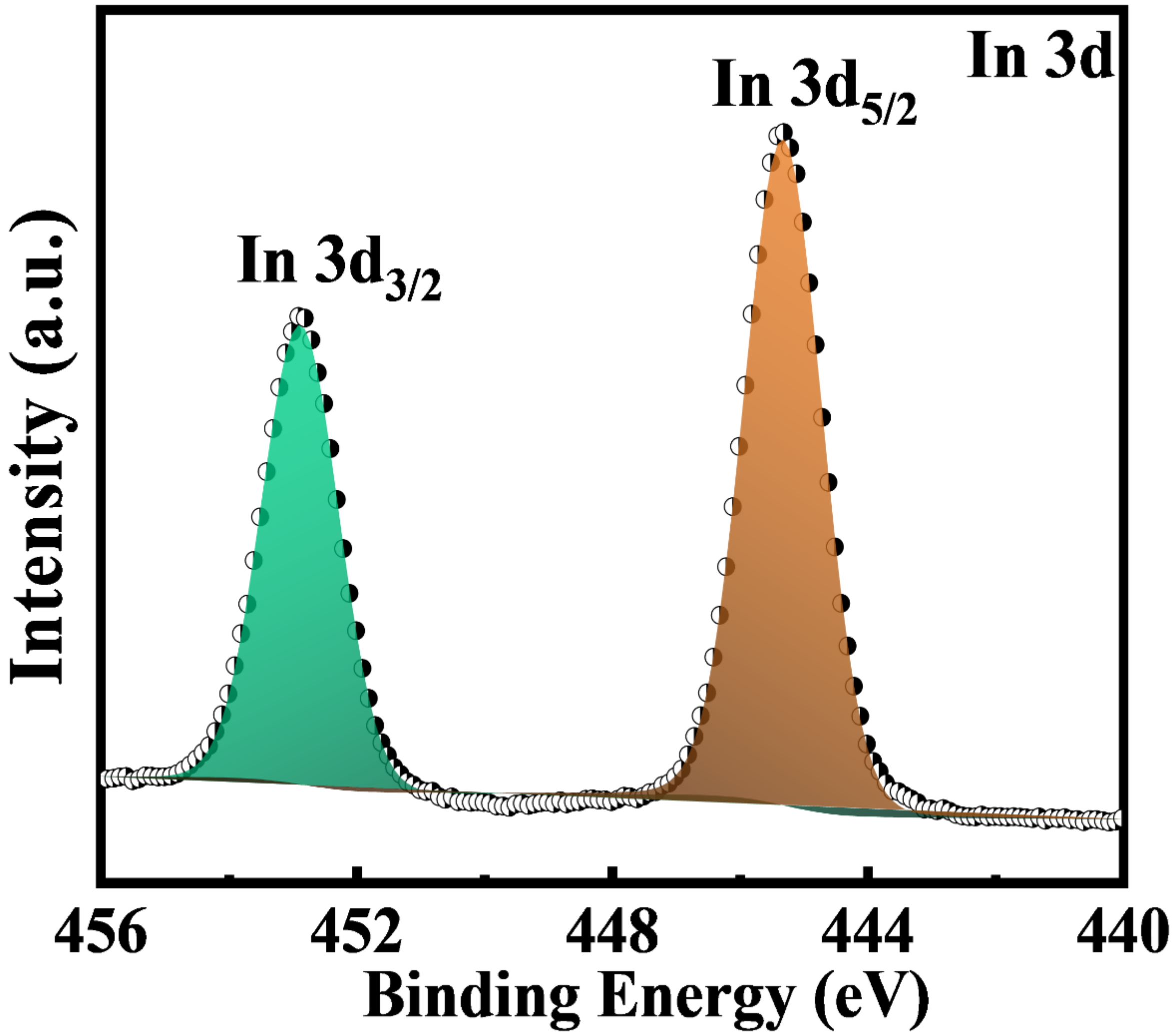

Figure S5. The In 3d XPS spectra of In-TCPP SURMOF.

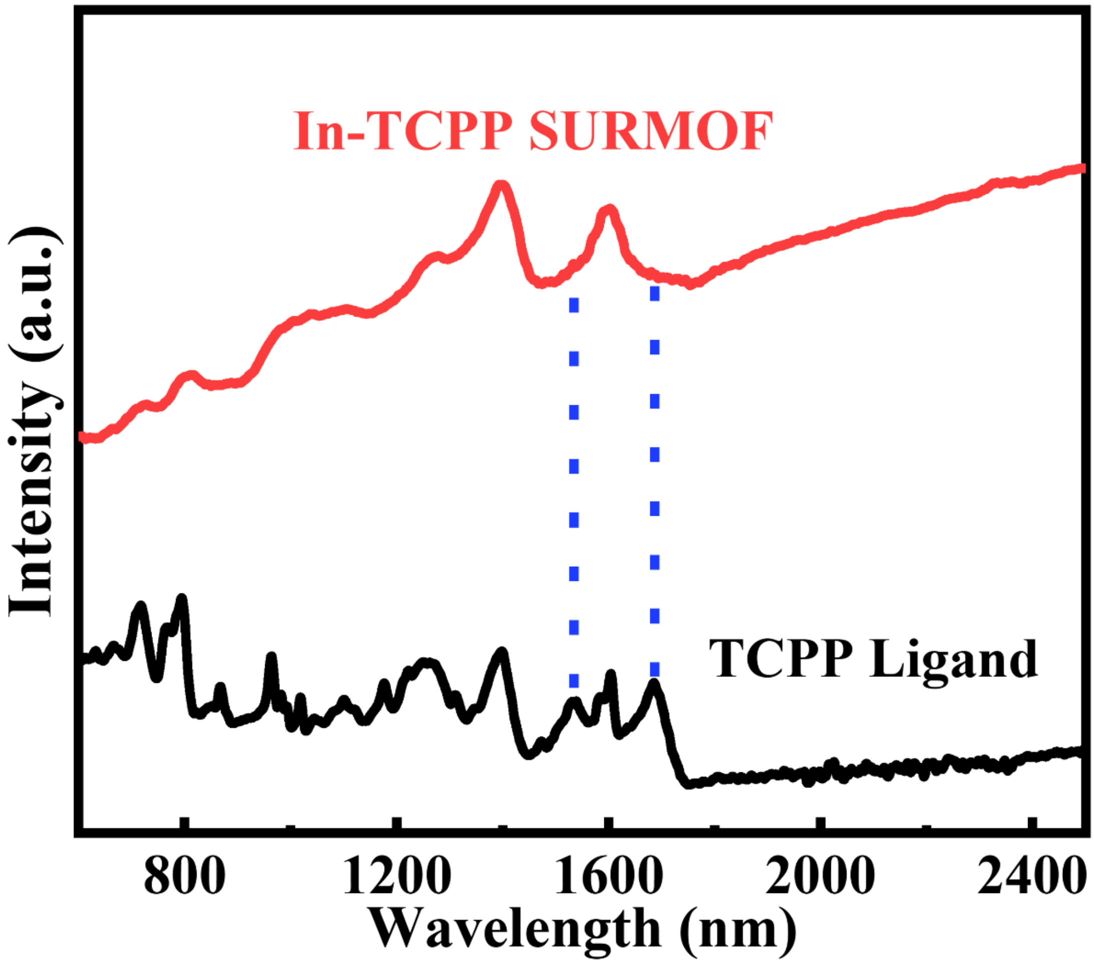

Figure S6. The IRRAS spectra of In-TCPP SURMOF and TCPP ligand.

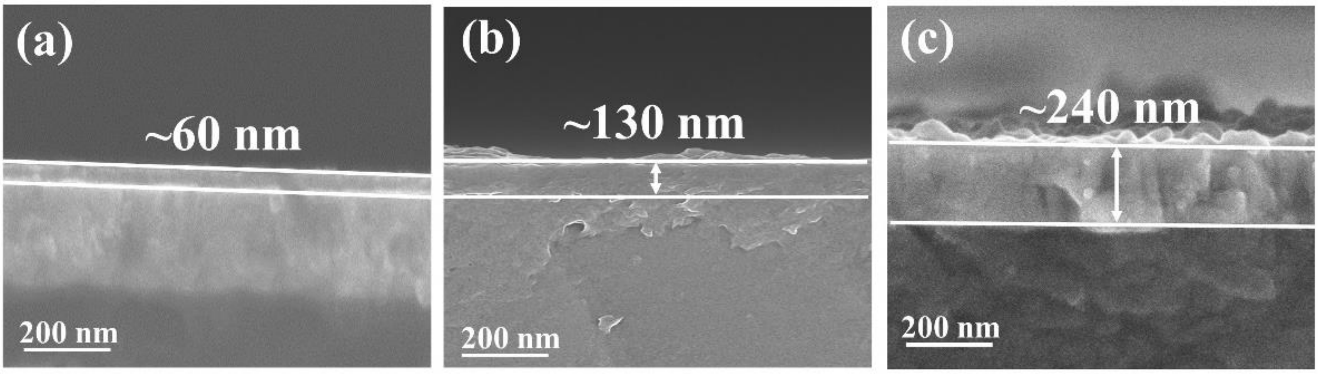

Figure S7. SEM images for different cycles of In-TCPP SURMOF on the SiO_2/Si substrate (a) 5 cycles, (b) 10 cycles and (c) 20 cycles.

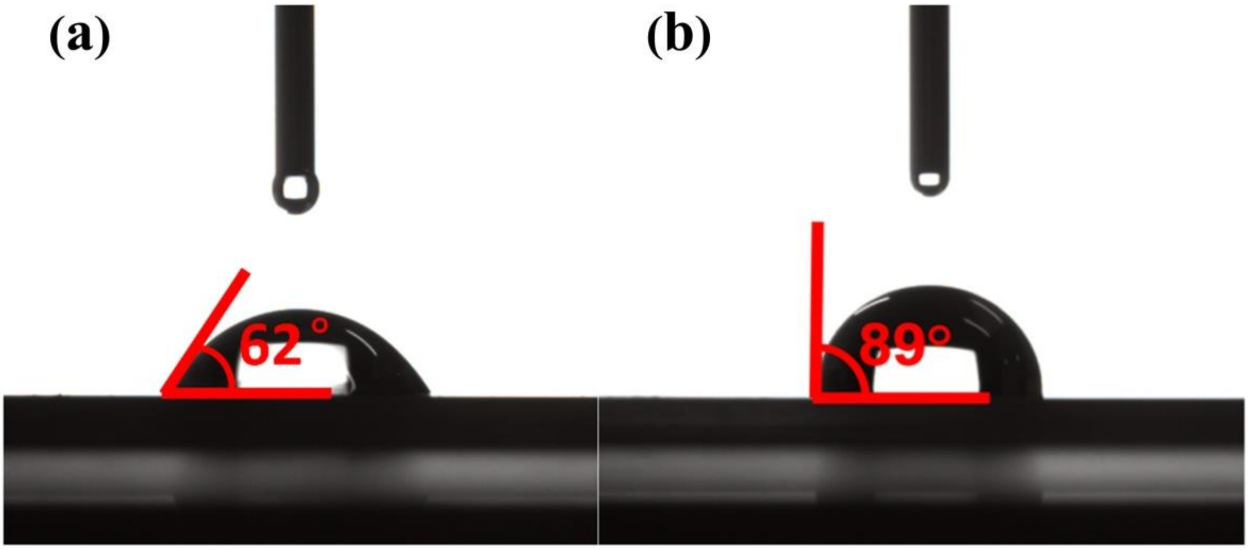

Figure S8. The contact angles of bare SiO_2/Si substrate (a) and In-TCPP SURMOF (b).

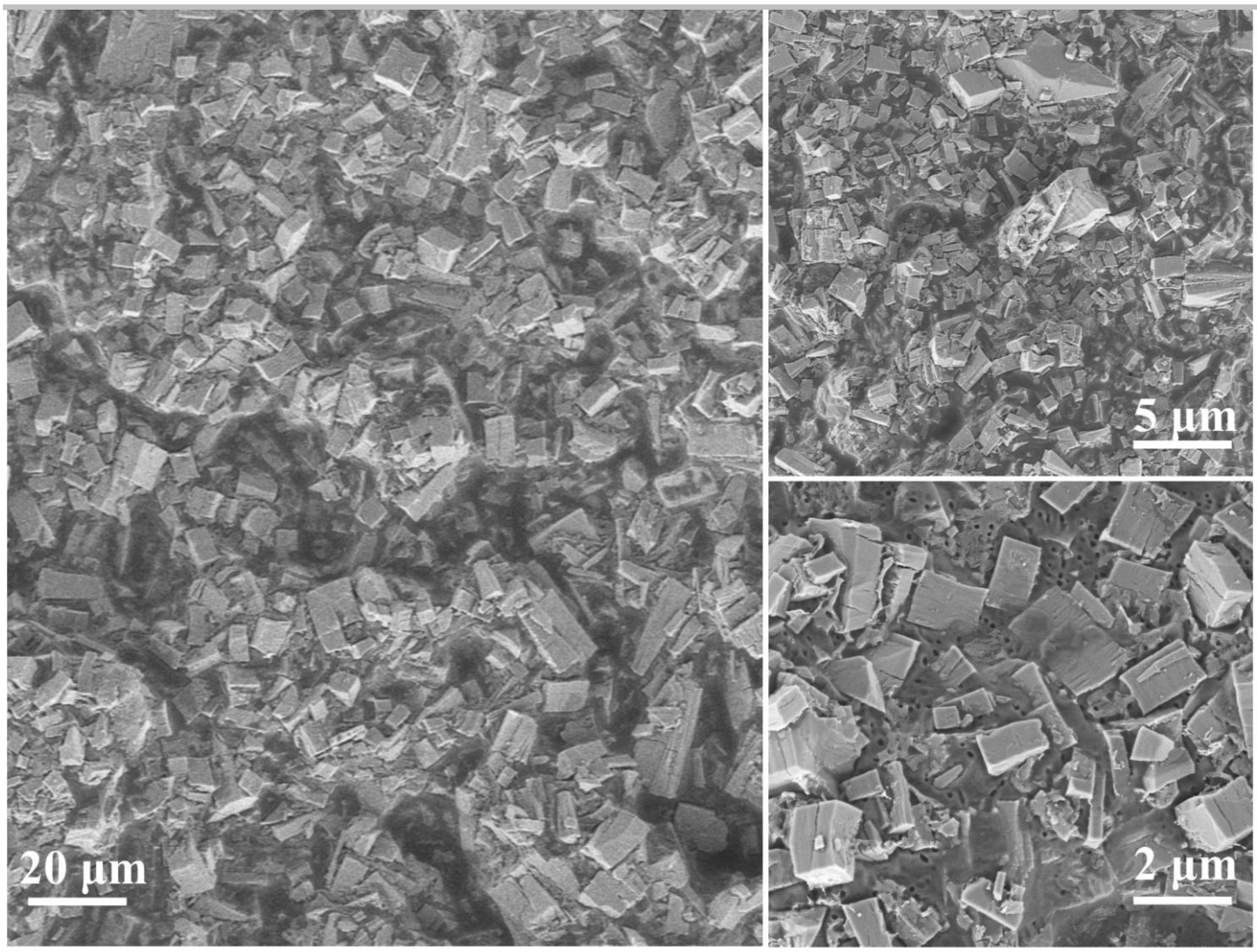

Figure S9. SEM images of mix-oriented In-TCPP thin film.

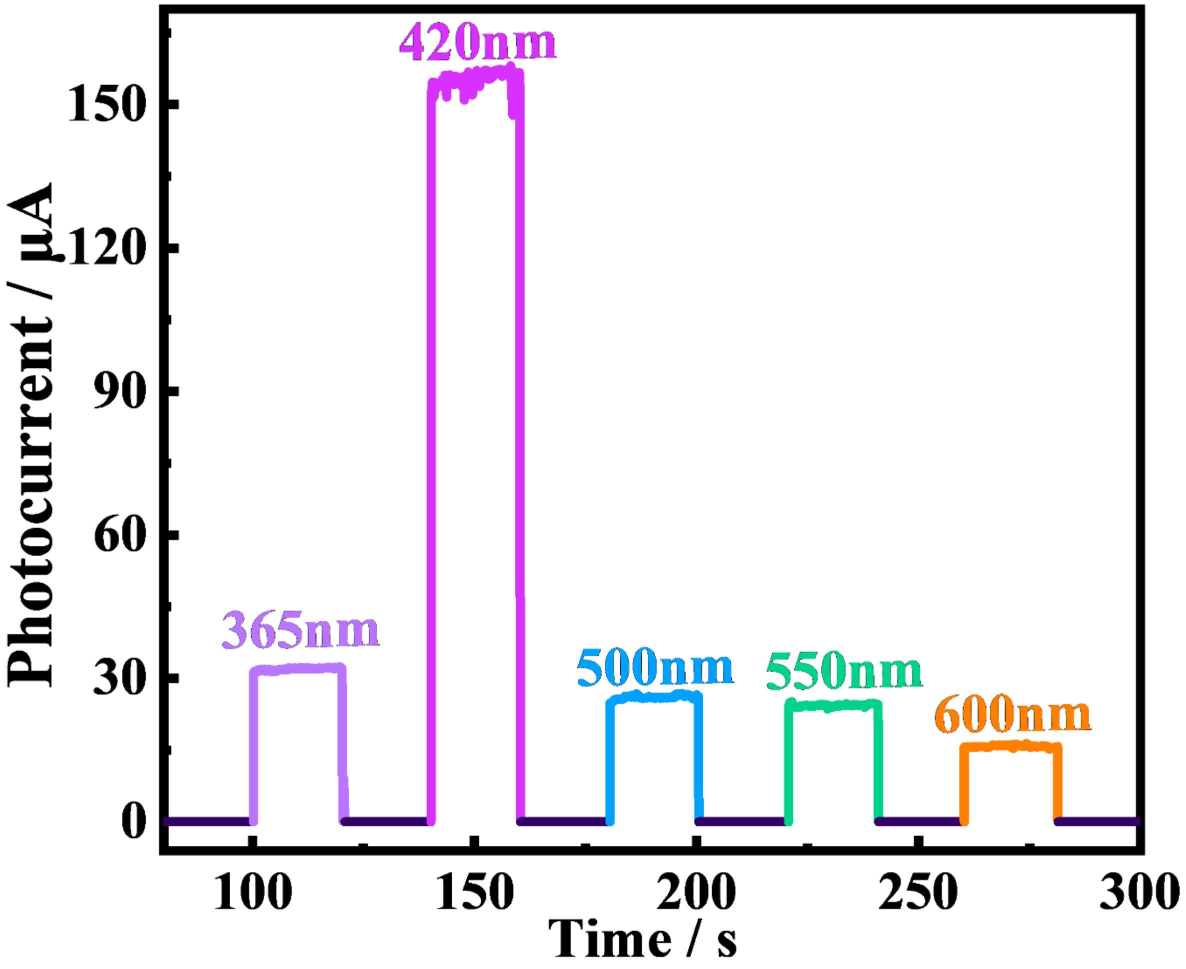

Figure S10. Wavelength-dependent photoresponse of In-TCPP SURMOF based photodetector.

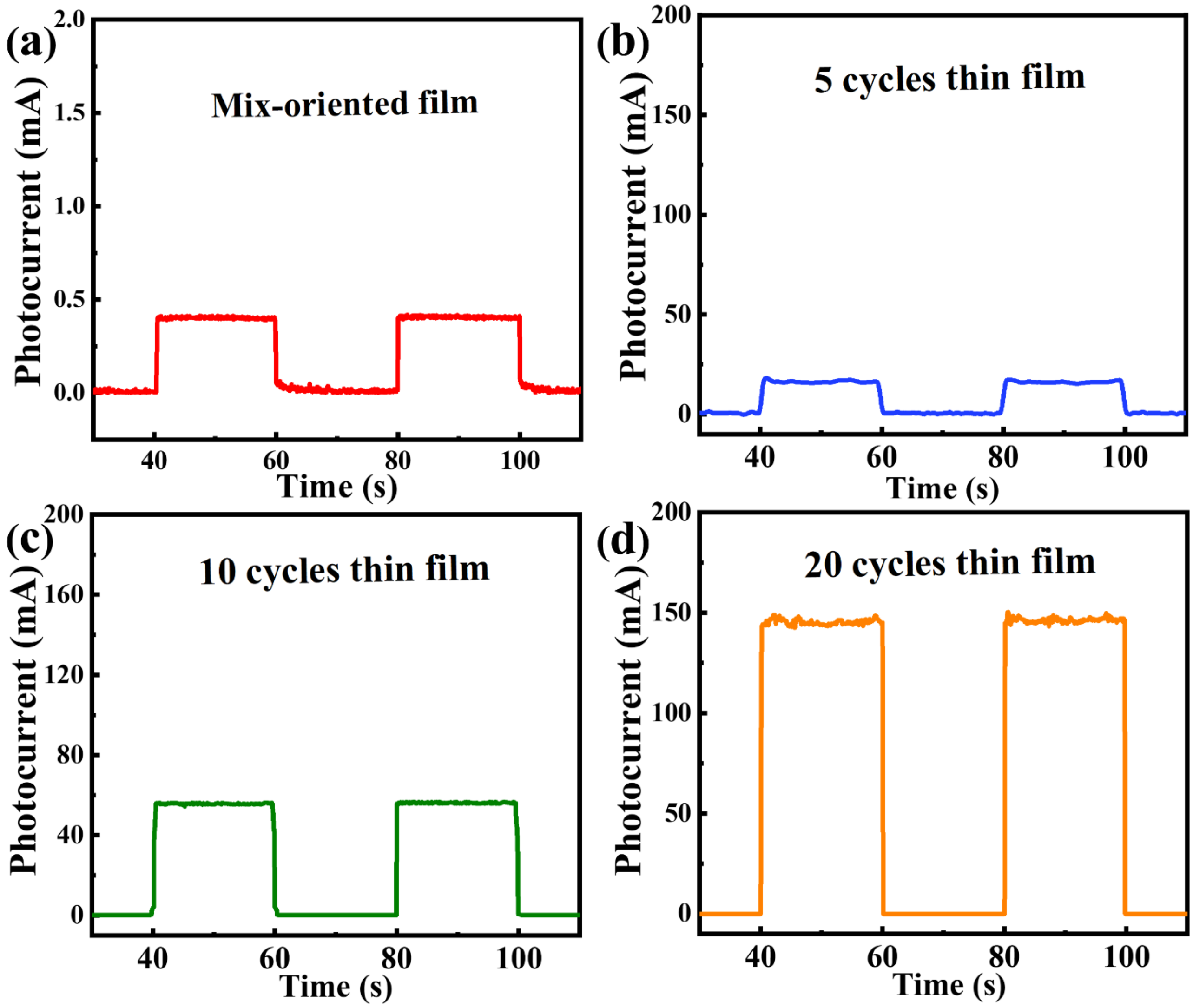

Figure S11. I-t curve of In-TCPP SURMOF with different thickness and mix-oriented thin film: (a) mix-oriented thin film, (b) 5 cycles, (c) 10 cycles and (d) 20 cycles

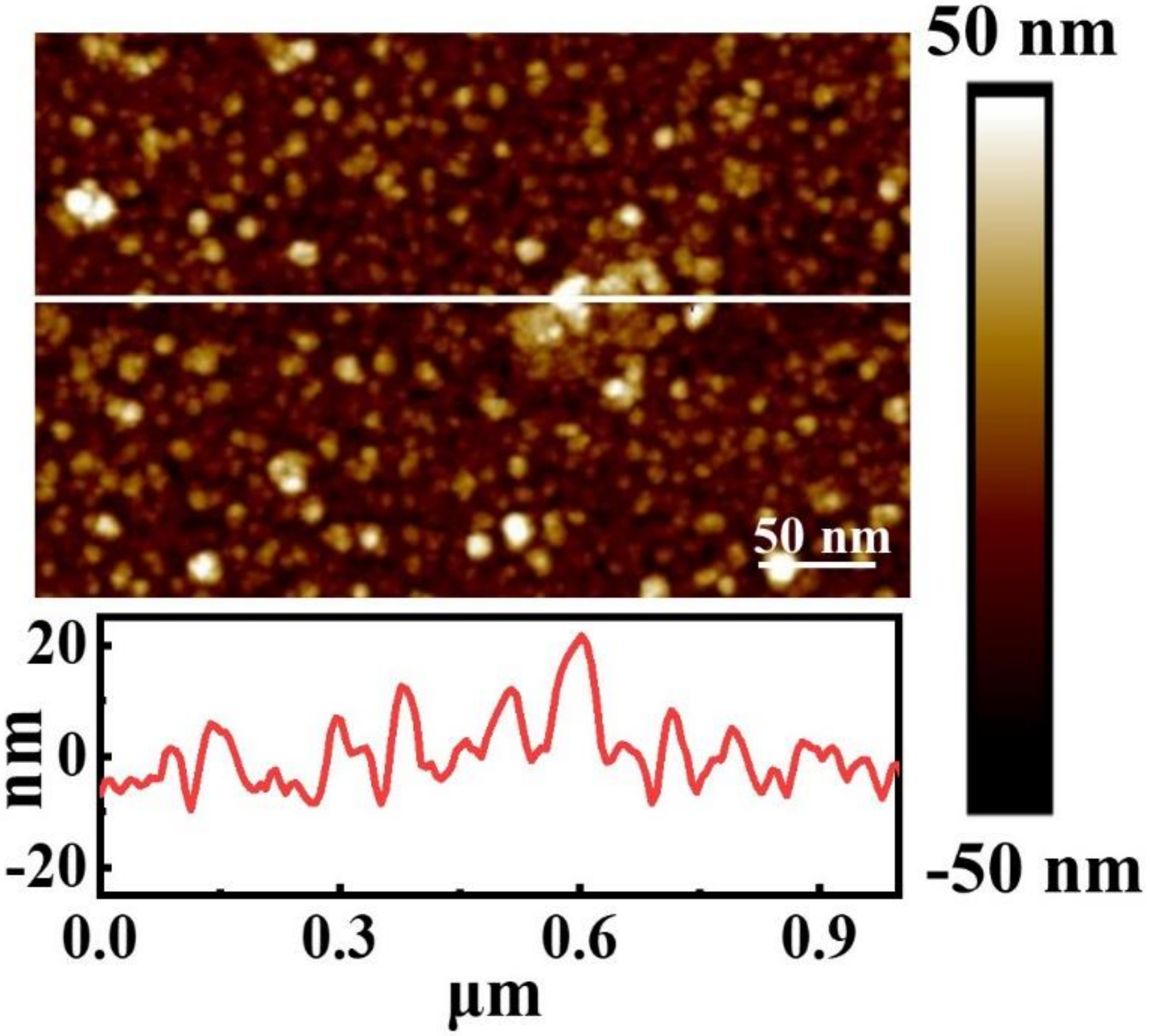

Figure S12. AFM image with roughness for In-TCPP SURMOF with 20 cycles.

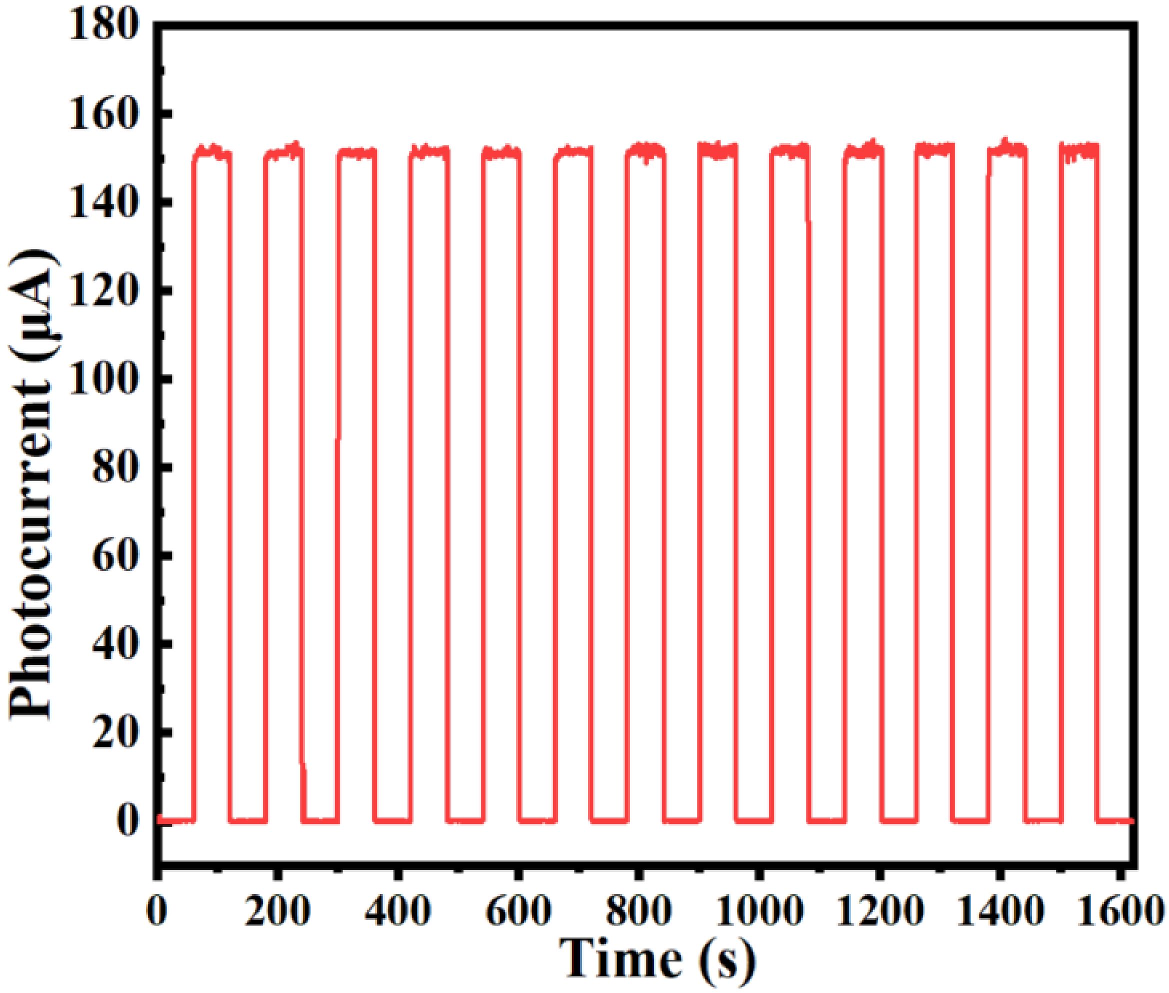

Figure S13. Time-resolved photocurrent stability test of light and dark current under 420 nm, each round of illumination time is 60 s.

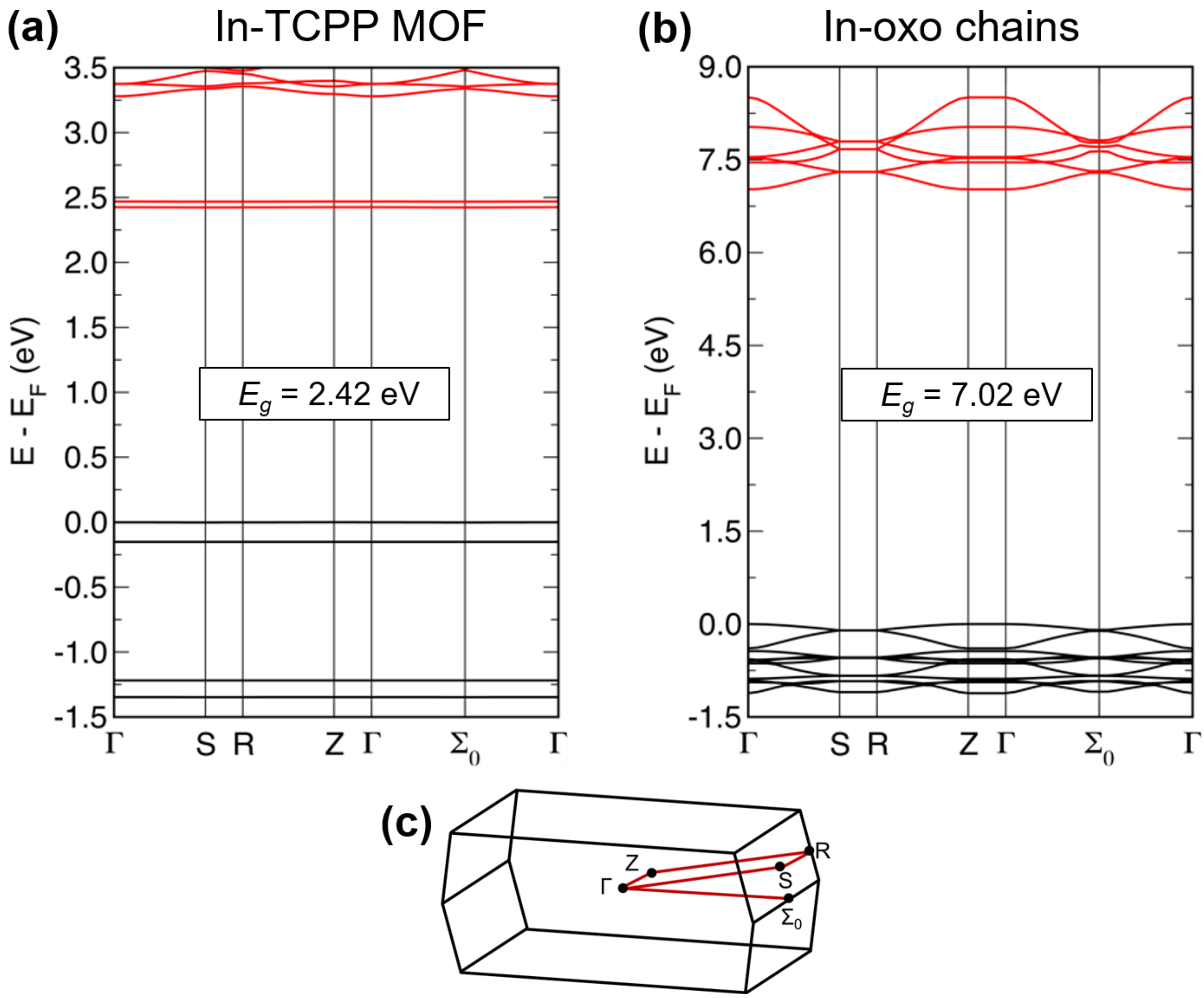

Figure S14. Band structure of In-TCPP MOF (a) and of In-oxo based chain model (b), and representation of the primitive Brillouin zone with the calculated path $\Gamma\rightarrow S\rightarrow R\rightarrow Z\rightarrow\Gamma\rightarrow\Sigma_0\rightarrow\Gamma$ (c). Calculations with Crystal17 at the HSE06-D3(BJ)/POB-TZVP//PBE-D3(BJ)/POB-TZVP level of theory.

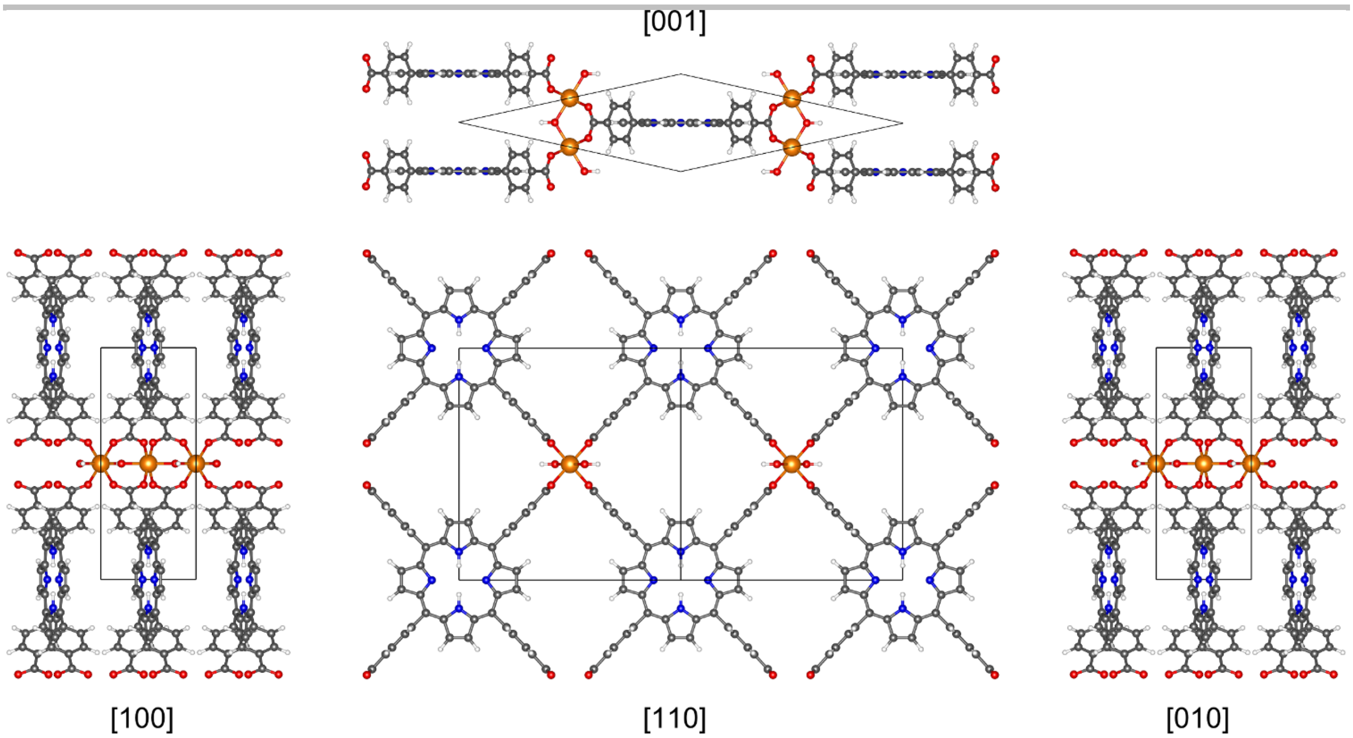

Figure S15. Atomistic representation of the In-TCPP MOF along the different crystallographic directions. The quadrilaterals indicate the unit cell used in the calculations. Color code: In orange, O red, N blue, C grey, H white.

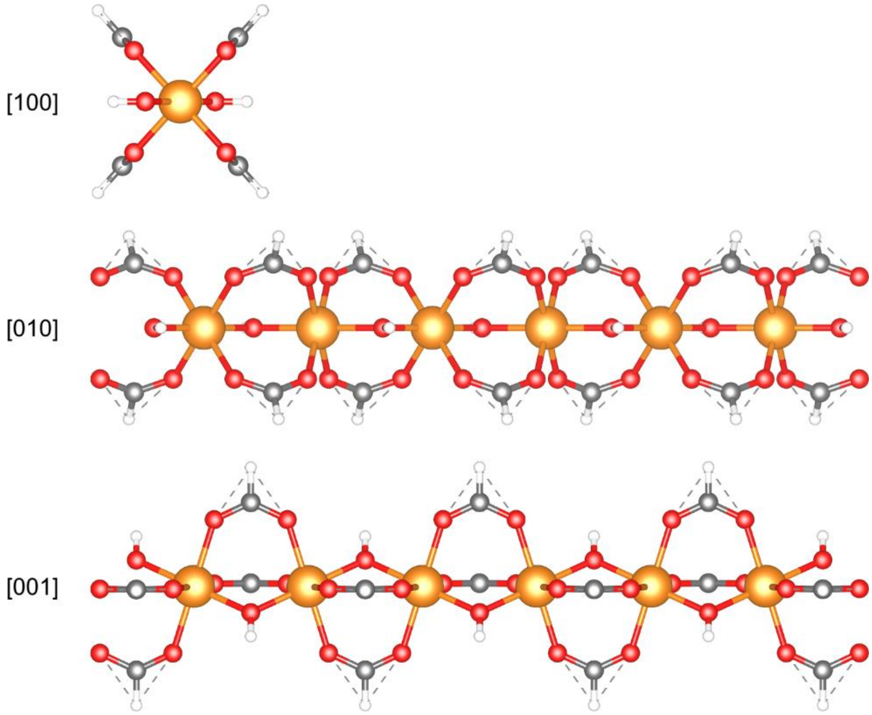

Figure S16. Atomistic representation of the In-oxo based chain model along the different crystallographic directions. Color code: In orange, O red, C grey, H white.

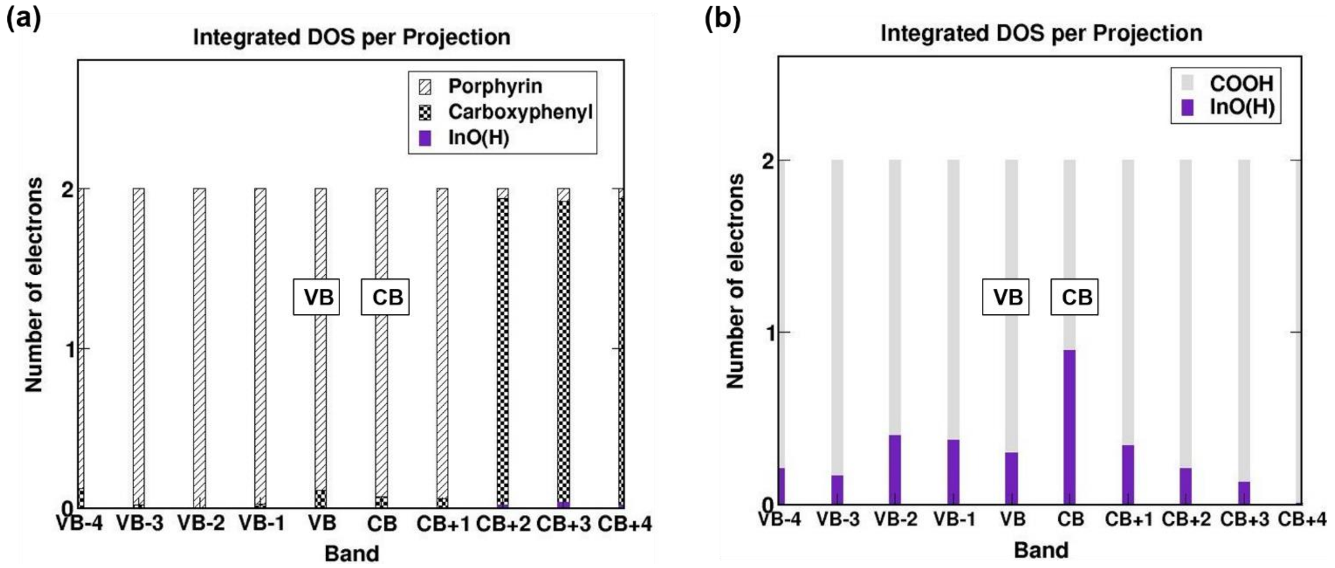

Figure S17. Integrated Density-of-States per projection (IDOS), as calculated for the In-TCPP MOF (a) and for the In-oxo based chain model (b). VB denotes valence band maximum, CB denotes conduction band minimum. Calculations with Crystal17 at the PBE-D3(BJ)/POB-TZVP//PBE-D3(BJ)/POB-TZVP level of theory.

Table S1. Comparison of the interlamellar distance of porphyrinic MOFs with face-to-face stacking.

**MOFs**	**Dimension**	**The distance (Å)**	**References**
SURMOF In-TCPP	3D	7.1	**This work**
SURMOF-2	2D	6.7	[11]
NaTCPP	2D	6.7	[12]
5•(BArF)$_8$	3D	8.2	[13]
Ar$_4$TCHP	3D	10.8	[14]
1•(BArF)$_8$	3D	11.8	[15]
ZnTCPP	3D	12.1	[16]
Zn^{II}-porphyrins	3D	14.1	[17]
PCN-224	3D	19.3	[18]
PCN-221	3D	19.4	[19]

Table S2. Comparison of characteristic parameters for state-of-the-art photodetectors.

Form	Devices structure	R [AW^{-1}]	D* [Jones]	Measurement conditions	References
MOFs	In-TCPP	30.8	7.28×10^{14}	λ = 420 nm,	This work
	$Cu_3(C_{18}H_6(NH)_6)_2$	0.3	3.2×10^{11}	λ = 450 nm	[20]
	$Fe_3(THT)_2(NH_4)_3$	0.004	7×10^{8}	λ = 785 nm	[21]
	Zn(TPP)			λ = 365-640 nm	[22]
Porphyrinic complexes	CS-DP and $PC_{71}BM$	0.33	5.73×10^{13}	λ = 850 nm	[23]
	DHTBTEZP and $PC_{61}BM$		10^{12}	λ = 380-960 nm	[24]
Organic compounds	C_{60} fibers	2.595	2.7×10^{12}	λ = 400 nm	[25]
	PbPc: C_{70}		2.7×10^{12}	λ = 400-1100 nm	[26]
	T3M3B7		3.05×10^{12}	λ = 540 nm	[27]
	PCPDTBT: $PC_{70}BM$	0.387	10^{13}	λ = 800 nm	[28]
	Y-TiOPc	2227	3.1×10^{14}	λ = 780 nm	[29]
Organic crystal	1,6-DTEP	2.86×10^{6}	1.49×10^{18}	λ = 370 nm	[30]
Perovskite	$CsPb_{0.922}Sn_{0.078}I_3$	1.18×10^{3}	6.43×10^{13}	λ = 300-480 nm	[31]
	Cu hybrid $MAPbI_3$	0.37	1.06×10^{12}	λ = 520 nm	[32]
	$CsPbCl_3$	0.16	10^{10}	λ = 280-400 nm	[33]
Inorganic complexes	CdTe	0.0006	1.0×10^{9}	λ = 473 nm	[34]
	$GaSe/VO_2$	0.358	2.14×10^{11}	λ = 404 nm	[35]
	$PtSe_2$/Perovskite	0.118	2.91×10^{12}	λ = 808 nm	[36]
	Graphene/GaN		1.0×10^{17}	λ = 325 nm	[37]

Table S3. Resistance of In-TCPP SURMOFs with different LPE cycles.

**LPE cycles**	5	10	15	20
**Resistance(Ω)**	1.08×10^{10}	5.88×10^{9}	4.55×10^{9}	3.03×10^{9}

Table S4. The roughness of In-TCPP SURMOF with 15 and 20 cycles recording by AFM.

**LPE cycles**	15	20
**Roughness**	~ ±5 nm	~ ±15 nm

References

[1] D.-J. Li, Z.-G. Gu, J. Zhang, *Chem. Sci.* **2020**, *11*, 1935.
[2] T. Sivakumar, R. Manavalan, K. Valliappan, *Acta Chromatogr.* **2007**, *19*, 29.
[3] O. Zybaylo, O. Shekhah, H. Wang, M. Tafipolsky, R. Schmid, D. Johannsmann, C. Wöll, *Phys. Chem. Chem. Phys.* **2010**, *12*, 8092.
[4] R. Dovesi, A. Erba, R. Orlando, C. M. Zicovich-Wilson, B. Civalleri, L. Maschio, M. Rérat, S. Casassa, J. Baima, S. Salustro, B. Kirtman, *WIREs Comput. Mol. Sci.* **2018**, *8*, 1360.
[5] M. F. Peintinger, D. V. Oliveira, T. Bredow, *J. Comput. Chem.* **2013**, *34*, 451.
[6] S. Grimme, J. Antony, S. Ehrlich, H. Krieg, *J. Chem. Phys.* **2010**, *132*, 154104.
[7] J. P. Perdew, K. Burke, M. Ernzerhof, *Phys. Rev. Lett.* **1996**, *77*, 3865.
[8] J. H. Luo, J. Wang, Y. Cao, S. Yao, L. R. Zhang, Q. S. Huo, Y. L. *Liu, Inorg. Chem. Front.* **2017**, *4*, 139.
[9] J. Heyd, G. E. Scuseria, M. Ernzerhof, *J. Chem. Phys.* **2003**, *118*, 8207.
[10] H. J. Monkhorst, J. D. Pack, *Phys. Rev. B* **1976**, *13*, 5188.
[11] S. M. Marschner, R. Haldar, O. Fuhr, C. Wöll, S. Bräse, *Chem. - Eur. J.* **2020**, *18*, 1390.
[12] Y. Diskin-Posner, I. Goldberg, *New J. Chem.* **2001**, *25*, 899.
[13] C. García-Simón, A. Monferrer, M. Garcia-Borràs, I. Imaz, D. Maspoch, M. Costas, X. Ribas, *Chem. Comm.* **2019**, *55*, 798.
[14] O. S. Finikova, A. V. Cheprakov, P. J. Carroll, S. Dalosto, S. A. Vinogradov, *Inorg. Chem.* **2002**, *41*, 6944.
[15] C. Fuertes-Espinosa, A. Gómez-Torres, R. Morales-Martínez, A. Rodríguez-Fortea, C. García-Simón, F. Gándara, I. Imaz, J. Juanhuix, D. Maspoch, J. M. Poblet, *Angew. Chem. Int. Ed.* **2018**, *130*, 11464.
[16] Y. Diskin-Posner, S. Dahal, I. Goldberg, *Chem. Comm.* **2000**, 585.
[17] C. García-Simón, M. Garcia-Borràs, L. Gómez, T. Parella, S. Osuna, J. Juanhuix, I. Imaz, D. Maspoch, M. Costas, X. Ribas, *Nat. Commun.* **2014**, *5*, 1.
[18] S. Yuan, J.-S. Qin, J. L. Li, L. Huang, L. Feng, Y. Fang, C. Lollar, J. D. Pang, L. L. Zhang, D. Sun, *Nat. Commun.* **2018**, *9*, 808.
[19] W. Morris, B. Volosskiy, S. Demir, F. Gándara, P. L. McGrier, H. Furukawa, D. Cascio, J. F. Stoddart, O. M. Yaghi, *Inorg. Chem.* **2012**, *51*, 6443.

[20] L.-A. Cao, M.-S. Yao, H.-J. Jiang, S. Kitagawa, X.-L. Ye, W.-H. Li, G. Xu, *J. Mater. Chem. A* **2020**, *8*, 9085.
[21] H. Arora, R. Dong, T. Venanzi, J. Zscharschuch, H. Schneider, M. Helm, X. L. Feng, E. Cánovas, A. Erbe, *Adv. Mater.* **2020**, *32*, 1907063.
[22] X. J. Liu, M. Kozlowska, T. Okkali, D. Wagner, T. Higashino, G. Brenner-Weiß, S. M. Marschner, Z. H. Fu, Q. Zhang, H. Imahori, S. Bräse, W. Wenzi, C. Wöll, L. Heinke. *Angew. Chem. Int. Ed.* **2019**, *58*, 9590.
[23] L. G. Xiao, S. Chen, X. B. Chen, X. B. Peng, Y. Cao, X. J. Zhu, *J. Mater. Chem. C* **2018**, *6*, 3341.
[24] L. S. Li, Y. Y. Huang, J. B. Peng, Y. Cao, X. B. Peng, *J. Mater. Chem. C* **2014**, *2*, 1372.
[25] K. Q. Liu, S. Gao, Z. Zheng, X. L. Deng, S. Mukherjee, S. S. Wang, H. Xu, J. F Wang, J. F. Liu, T. Y. Zhai, Y. Fu, *Adv. Mater.* **2019**, *31*, 1808254.
[26] Z. S. Su, F. H. Hou, X. Wang, Y. Gao, F. M. Jin, G. Zhang, Y. T. Li, L. G. Zhang, B. Chu, W. L. Li, *ACS Appl. Mater. Interfaces* **2015**, *7*, 2529.
[27] D.-S. Leem, K.-H. Lee, K.-B. Park, S.-J. Lim, K.-S. Kim, Y. W. Jin, S. Lee, *Appl. Phys. Lett.* **2013**, *103*, 1321.
[28] X. Gong, M.-H. Tong, S. H. Park, M. Liu, A. Jen, A. J. Heeger, *Sensors* **2010**, *10*, 6488.
[29] X. L. Li, S. R. Wang, Y. Xiao, X. G. Li, *J. Mater. Chem. C* **2016**, *4*, 5584.
[30] J. W. Tao, D. Liu, Z. S. Qin, B. Shao, J. B. Jing, H. X. Li, H. L. Dong, B. Xu, W. J. Tian, *Adv. Mater.* **2020**, *32*, 1907791.
[31] Z. T. Du, D. F. Fu, T. Yang, Z. Fang, W. N, Liu, F. M. Gao, L. Wang, Z. B. Yang, J. Teng, H. Zhang, W. Y. Yang, *J. Mater. Chem. C* **2018**, *6*, 6287.
[32] Z. Y. Liu, X. Y. Liu, B. Sun, X. H. Tan, H. B. Ye, J. X. Zhou, Z. R. Tang, T. L. Shi, G. L. Liao, *Adv. Mater. Technol.* **2020**, *5*, 2000260.
[33] Z.Y. Rao, W. Y. Liang, H. M. Huang, J. Ge, W. L. Wang, S. S. Pan, *Opt. Mater. Express* **2020**, *10*, 1374.
[34] R. Q. Cheng, Y. Wen, L. Yin, F. M. Wang, F. Wang, K. L. Liu, T. A. Shifa, J. Li, C. Jiang, Z. X. Wang, *Adv. Mater.* **2017**, *29*, 1703122.
[35] J. Y. Zhou, M. Z. Xie, H. Ji, A. Y. Cui, Y. Ye, K. Jiang, L. Y. Shang, J. Z. Zhang, Z. G. Hu, J. H. Chu, *ACS Appl. Mater. Interfaces* **2020**, *12*, 18674.
[36] Z.-X. Zhang, L.-H. Zeng, X.-W. Tong, Y. Gao, C. Xie, Y. H. Tsang, L.-B. Luo, Y.-C. Wu, *J. Phys. Chem. Lett.* **2018**, *9*, 1185.
[37] J. Li, X. Xi, S. Lin, Z. H. Ma, X. D. Li, L. X. Zhao, *ACS Appl. Mater. Interfaces* **2020**, *12*, 11965.
